# Large-scale production and protein engineering of G protein-coupled receptors for structural studies

**DOI:** 10.3389/fphar.2015.00066

**Published:** 2015-03-31

**Authors:** Dalibor Milić, Dmitry B. Veprintsev

**Affiliations:** ^1^Laboratory of Biomolecular Research, Paul Scherrer Institut, VilligenSwitzerland; ^2^Department of Biology, Eidgenössische Technische Hochschule Zürich, ZürichSwitzerland

**Keywords:** G protein-coupled receptor (GPCR), expression, purification, protein engineering, isotope labeling

## Abstract

Structural studies of G protein-coupled receptors (GPCRs) gave insights into molecular mechanisms of their action and contributed significantly to molecular pharmacology. This is primarily due to technical advances in protein engineering, production and crystallization of these important receptor targets. On the other hand, NMR spectroscopy of GPCRs, which can provide information about their dynamics, still remains challenging due to difficulties in preparation of isotopically labeled receptors and their low long-term stabilities. In this review, we discuss methods used for expression and purification of GPCRs for crystallographic and NMR studies. We also summarize protein engineering methods that played a crucial role in obtaining GPCR crystal structures.

## Introduction

G protein-coupled receptors are 7TM proteins that transmit signals between the cellular environment and its interior. Due to their key role in pathophysiology, GPCRs are important drug targets (Roth, 2005). One of the features of GPCRs and proteins involved in their signaling pathways is that they are very dynamic and flexible proteins and change their conformation during the signal transduction. Binding of ligands stabilizes particular conformations of a GPCR, which result in the activation of G proteins (Hepler and Gilman, 1992), G protein-coupled kinases (Gurevich et al., 2012) and arrestin-mediated (Shukla et al., 2011) signaling pathways. The efficacy of the ligands varies from agonists, which activate a specific response, to antagonists, which block the binding site without changing the basal activity, to inverse agonists, which further reduce the activity below the basal level. Some ligands show bias, preferentially activating either G protein or arrestin pathways, or even changing the balance between the activation of different G protein subtypes (Mary et al., 2013; Nygaard et al., 2013; Venkatakrishnan et al., 2013). This rich pharmacology offers a large therapeutic potential, and understanding the underlying structural basis may help in the rational design of new drugs (Congreve et al., 2014).

**FIGURE 1:**
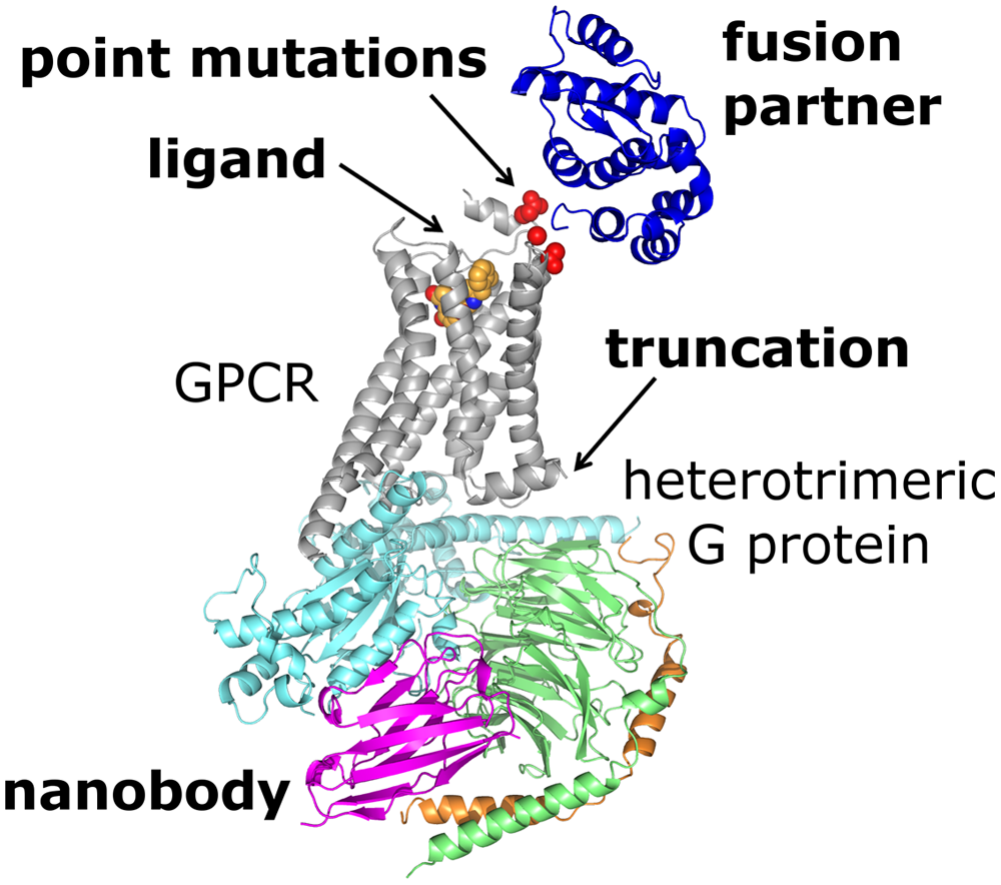
**Crystal structure of the β_**2**_-adrenoceptor–G_**s**_ protein complex (PDB ID: 3SN6) illustrates some of the protein engineering strategies available for structural studies of GPCRs.** The receptor (gray) was N-terminally fused with T4L (blue) and complexed with an agonist (orange spheres). The complex with G_s_ protein (α-subunit shown in cyan, β-subunit in green and γ-subunit in orange) is additionally stabilized by nanobody (magenta). Three point mutations (residues shown as red spheres) had to be introduced in order to delete a glycosylation site (mutation N187E) and to increase expression level of the T4L–β_2_-adrenoceptor chimera (mutations M96T, M98T). Finally, the receptor molecule was truncated to remove a flexible C-terminal tail interfering with crystallization.

In the last several years, X-ray crystallography gave significant contribution to understanding of molecular mechanisms of GPCR signaling. Until the end of 2014, crystal structures of the transmembrane regions have been published for 29 unique GPCRs (**Table 1**). This terrific progress is due to several technological advances in GPCR crystallization (**Figure 1**), including use of antibodies, in particular nanobodies (Steyaert and Kobilka, 2011), and/or fusion partners for conformational stabilization and an increase in hydrophilic molecular surface, truncations and deletions of flexible loops to reduce molecular flexibility, as well as an introduction of stabilizing point mutations in order to increase thermostability of a detergent-solubilized receptor (Tate and Schertler, 2009; Tate, 2012). Also, this progress can partially be attributed to an increased use of LCP as a crystallization environment particularly suitable for membrane proteins (Caffrey and Cherezov, 2009) along with development of synchrotron X-ray microfocus beamlines and, more recently, X-ray free-electron lasers (Liu et al., 2013; Liu et al., 2014; Weierstall et al., 2014).

**Table 1:** G protein-coupled receptors with experimentally determined structure of a 7TM domain (published until the end of 2014).

GPCR*^a^*	Source organism*^b^*	GPCR family (Class)*^a^*	Expression system	References*^c^*
**Isolation from natural source**				
Rhodopsin	Bovine	Opsin (A)	Bovine (rod photoreceptor cells)	Palczewski et al. (2000), Teller et al. (2001), Okada et al. (2002, 2004), Li et al. (2004), Nakamichi and Okada (2006), Salom et al. (2006b), Nakamichi et al. (2007), Park et al. (2008, 2013), Scheerer et al. (2008), Makino et al. (2010), Choe et al. (2011), Szczepek et al. (2014)
Rhodopsin	Squid	Opsin (A)	Squid (rhabdomeric photoreceptor cells)	Murakami and Kouyama (2008, 2011)
				
**Expression in mammalian cells**				
Rhodopsin	Bovine	Opsin (A)	COS-1	Standfuss et al. (2007)
Rhodopsin	Bovine	Opsin (A)	HEK293S(TetR) GnTI^-^	Standfuss et al. (2011), Deupi et al. (2012), Singhal et al. (2013)
**Expression in insect cells**				
β_1_-adrenoceptor	Turkey	Adrenoceptors (A)	High Five	Warne et al. (2008, 2011, 2012), Moukhametzianov et al. (2011), Christopher et al. (2013), Huang et al. (2013), Miller-Gallacher et al. (2014)
β_2_-adrenoceptor	Human	Adrenoceptors (A)	*Sf*9	Cherezov et al. (2007), Rasmussen et al. (2007, 2011a,b), Hanson et al. (2008), Bokoch et al. (2010), Wacker et al. (2010), Rosenbaum et al. (2011), Zou et al. (2012), Ring et al. (2013), Weichert et al. (2014)
D_3_ receptor	Human	Dopamine (A)	*Sf*9	Chien et al. (2010)
M_2_ receptor	Human	Acetylcholine (muscarinic) (A)	*Sf*9	Haga et al. (2012), Kruse et al. (2013)
M_3_ receptor	Rat	Acetylcholine (muscarinic) (A)	*Sf*9	Kruse et al. (2012), Thorsen et al. (2014)
5-HT_1B_ receptor	Human	5-Hydroxytryptamine (A)	*Sf*9	Wang et al. (2013a)
5-HT_2B_ receptor	Human	5-Hydroxytryptamine (A)	*Sf*9	Liu et al. (2013), Wacker et al. (2013)
A_2A_ receptor	Human	Adenosine (A)	*Sf*9	Jaakola et al. (2008), Doré et al. (2011), Xu et al. (2011b), Congreve et al. (2012), Liu et al. (2012b)
A_2A_ receptor	Human	Adenosine (A)	High Five	Lebon et al. (2011b)
P2Y_12_ receptor	Human	P2Y (A)	*Sf*9	Zhang et al. (2014a,b)
CXCR4	Human	Chemokine (A)	*Sf*9	Wu et al. (2010)
CCR5	Human	Chemokine (A)	*Sf*9	Tan et al. (2013)
δ receptor	Mouse	Opioid (A)	*Sf*9	Granier et al. (2012)
δ receptor	Human	Opioid (A)	*Sf*9	Fenalti et al. (2014)
κ receptor	Human	Opioid (A)	*Sf*9	Wu et al. (2012)
μ receptor	Mouse	Opioid (A)	*Sf*9	Manglik et al. (2012)
NOP receptor	Human	Opioid (A)	*Sf*9	Thompson et al. (2012)
NTS_1_ receptor	Rat	Neurotensin (A)	High Five	White et al. (2012)
PAR1	Human	Proteinase-activated (A)	*Sf*9	Zhang et al. (2012)
OX_2_	Human	Orexin (A)	*Sf*9	Yin et al. (2015)
S1P_1_ receptor	Human	Lysophospholipid (S1P) (A)	*Sf*9	Hanson et al. (2012)
FFA1 receptor	Human	Free fatty acid (A)	*Sf*9	Srivastava et al. (2014)
CRF_1_ receptor	Human	Corticotropin-releasing factor (B)	High Five	Hollenstein et al. (2013)
Glucagon receptor	Human	Glucagon (B)	*Sf*9	Siu et al. (2013)
mGlu_1_ receptor	Human	Metabotropic glutamate (C)	*Sf*9	Wu et al. (2014)
mGlu_5_ receptor	Human	Metabotropic glutamate (C)	*Sf*21	Doré et al. (2014)
SMO	Human	Frizzled (F)	*Sf*9	Wang et al. (2013b, 2014), Weierstall et al. (2014)
**Expression in yeast**				
H_1_ receptor	Human	Histamine (A)	*P. pastoris* SMD1163	Shimamura et al. (2011)
A_2A_ receptor	Human	Adenosine (A)	*P. pastoris* SMD1163	Hino et al. (2012)
**Expression in bacteria**				
CXCR1*^d^*	Human	Chemokine (A)	*E. coli* BL21(DE3)	Park et al. (2012b)
NTS_1_ receptor	Rat	Neurotensin (A)	*E. coli* BL21(DE3)	Egloff et al. (2014b)

*^a^*G protein-coupled receptor nomenclature used in this paper is according to the International Union of Basic and Clinical Pharmacology, IUPHAR (Alexander et al., 2013). *^b^*Binomial names of organisms: Bovine – *Bos taurus*; Human – *Homo sapiens*; Mouse – *Mus musculus*; Rat – *Rattus norvegicus*; Squid – *Todarodes pacificus*; Turkey – *Meleagris gallopavo*. *^c^*Publications reporting the structures. *^d^*The structural model is based on solid-state NMR spectroscopy.

Structures of several GPCRs were obtained in both inactive and active conformations in complexes with diverse sets of ligands, ranging from inverse agonists to full agonists (Maeda and Schertler, 2013). This gave us some basic understanding of ligand specificity and ligand-induced conformational changes in the studied receptors and their interactions with intracellular signaling partners (Granier and Kobilka, 2012; Venkatakrishnan et al., 2013). Furthermore, the crystal structures enabled structure-based fragment screening for this important superfamily of druggable proteins (Kolb et al., 2009; Carlsson et al., 2010, 2011; de Graaf et al., 2011; Christopher et al., 2013). In addition, we were able to grasp structural basis of disease-causing mutations in the visual GPCR rhodopsin for the first time (Singhal et al., 2013).

Despite the fantastic progress in the crystallography of GPCRs, several key questions about the functioning of the receptors, such as the basis for the ligand selectivity, the activation mechanism and the structural basis for biased signaling, remained open because these processes are likely to be determined not only by the structure but also by the dynamics of the system. NMR spectroscopy is an ideal method to provide such dynamic information.

In this paper we give an in-depth overview of strategies and methods used to prepare GPCRs for crystallization and NMR spectroscopy. Although soluble GPCR domains, like N-terminal extracellular domains of class B, C, and F GPCRs, have been structurally studied (e.g., Kunishima et al., 2000; Dann et al., 2001; Tsuchiya et al., 2002; Rana et al., 2013; Dong et al., 2014), we limit ourselves only to discussion of expression, purification and protein engineering of GPCR molecular entities comprising a 7TM domain.

## Expression

### Natural Sources

Because of its abundance in retina of eye, rhodopsin could be isolated from natural sources in relatively large amounts and used for structural studies. Bovine (*Bos taurus*) rhodopsin has been isolated from ROS membranes, where it comprises more than 90% of membrane proteins (Okada et al., 1998). This allowed extensive crystallization screening in order to find the optimal crystallization conditions (Okada et al., 2000; Edwards et al., 2004; Salom et al., 2006a) resulting in crystal structures of several different forms of bovine rhodopsin (**Table 1**). Similarly, squid (*Todarodes pacificus*) rhodopsin was isolated from rhabdomic microvillar membranes in squid retina and its structure was determined in three different forms by X-ray crystallography (Murakami and Kouyama, 2008, 2011; Shimamura et al., 2008).

Bovine and squid rhodopsins are exceptional examples. The majority of GPCRs are present in scarce amounts in cell membranes, so in such cases purification from natural sources for structural studies would become very impractical and expensive. Moreover, modifications of GPCR molecules are usually needed for structural studies. Such modifications are impossible or very difficult to make in GPCRs isolated from natural sources. All these reasons make heterologous expression a preferred method of GPCR production (**Table 1**).

### Mammalian Cells

As many other eukaryotic proteins, GPCRs are heavily post-translationally modified. These modifications include glycosylation, fatty acylation (most often palmitoylation) and phosphorylation. *N*-linked glycosylation plays a role in proper GPCR folding in the endoplasmatic reticulum and subsequent trafficking to the plasma membrane. Phosphorylation is important in GPCR desensitization and internalization, while palmitoylation stabilizes receptor conformation in the membrane and thus might have a role in GPCR oligomerization and signaling. Because they have all necessary cellular and enzymatic machinery for correct post-translational processing, folding and insertion into a membrane, cultured mammalian cells are in general optimal systems for heterologous expression of functional mammalian GPCRs. Furthermore, lipidic content of mammalian membranes provides native environment for mammalian GPCRs. That does not mean that mammalian expression systems always provide enough protein suitable for structural studies. It often happens that the protein sample produced by overexpression is heterogenously glycosylated or that some portion of protein, although produced, is not properly folded and thus not functional.

There are two general ways for expressing proteins in mammalian cells: transient and stable expression (reviewed in Andréll and Tate, 2013). Mammalian cells can be transiently transfected by using recombinant non-replicative viruses with a GPCR gene (e.g., Semliki Forest Virus expression system) or by using chemical reagents, like cationic compounds, which form complexes with plasmid DNA and thus enable its insertion into a cell. In transient transfection the gene of interest does not integrate into the genome. On the contrary, stable transfection denotes integration of the gene of interest into the cellular genome. A gene for antibiotic resistance is also present in the same cloning cassette as the gene of interested, so the stable cells can be selected for antibiotic resistance. Incorporation into the genome can be random or specific by using a recombinase and site-specific recombination (e.g., Flp-In system from Life Technologies for production of adherent stable mammalian cells). In case of random incorporation, it is advisable to select clones with the highest functional expression levels. Otherwise, in the mixed population expression levels can drop over time, because the clones which express badly or not at all might grow faster. Alternatively, cells can be enriched for the highest expressers with FACS (Mancia et al., 2004; Thomas and Tate, 2014). With stable cell lines there is no need to transfect or infect the cells every time one produces the protein. This is certainly an advantage in comparison to transient expression.

Another important parameter to consider in heterologous expression is the choice of promoter. Constitutive promoters are not a good choice if the produced protein is toxic to cells. On the other hand, inducible promoters allow induction of expression after the cells have reached a certain density and minimize the negative effect of protein overexpression on the cells.

The first crystal structure of GPCR produced by heterologous expression was that of the thermally stable bovine rhodopsin mutant with an engineered disulfide bond (Standfuss et al., 2007). It was produced in adherent mammalian COS-1 cells transiently transfected using diethylaminoethyl-dextran. In this case, the rhodopsin gene was under control of constitutive adenovirus major late promoter (Oprian et al., 1987). From 50 transiently transfected 15-cm plates, Standfuss et al. (2007) got 2.5 mg rhodopsin 72 h after transfection, which resulted in 0.6 mg pure protein.

In order to avoid effects of heterogeneous and incomplete glycosylation in overexpression, Khorana’s group developed suspension adapted HEK293S(TetR) GnTI^-^ cells with a tetracycline-inducible expression system under control of cytomegalovirus promoter (Reeves et al., 2002a). Lacking the *N*-acetylglucosaminyltransferase I activity, these cells express proteins with *N*-linked glycosylation restricted to Man_5_GlcNAc_2_ *N*-glycan. Khorana and colleagues also showed that addition of sodium butyrate, a histone deacetylase inhibitor is beneficial for expression levels of rhodopsin (Reeves et al., 2002b). Stable HEK293S(TetR) GnTI^-^ cells were used in crystallographic studies of bovine rhodopsin mutants (Standfuss et al., 2011; Deupi et al., 2012; Singhal et al., 2013). For these studies, the cells in suspension were grown in 10 L wave bioreactors, which yielded ∼ 0.5 mg purified protein per liter of growth medium. Although originally used for expression of rhodopsin, HEK293S(TetR) GnTI^-^ cells proved to be useful for expression of several human membrane proteins, including a handful of GPCRs (reviewed in Andréll and Tate, 2013). Based on that, we believe this and some other mammalian cell lines will gain more prominence in future structural studies of GPCRs.

Although very useful in producing milligram amounts of functional GPCRs, expression in mammalian cells is accompanied with some drawbacks. In general, media and antibiotics used for GPCR production in mammalian cells are among the most expensive ones. Generation of stable cell lines is time consuming. It usually takes 3 weeks to obtain bulk, polyclonal sample of stable cells. Additional three to 6 weeks are needed to do clonal selection (Chaudhary et al., 2012). Alternatively, it can take up to 2 months to do several rounds of FACS until one gets polyclonal cells enriched for the best expressers and with a constant level of expression. On the other hand, transient expression is faster, but it is associated with difficulties in scaling up and higher costs. Nevertheless, transient transfection is very useful in initial screening of many GPCR constructs in adherent mammalian cells, including HEK293T cells that can replicate plasmids with SV40 origin of replication and thus increase expression levels.

### Insect Cells

Insect cells are the most common expression system used in crystallographic studies of GPCRs giving milligram amounts of pure protein per liter of cell culture. The majority of those receptors were expressed in *Spodoptera frugiperda Sf*9 cells (**Table 1**). *Trichoplusia ni* (*Tni*) High Five cells were used for production of turkey β_1_-adrenoceptor (Warne et al., 2003, 2008, 2009), rat neurotensin NTS_1_ receptor (White et al., 2012) and human corticotropin-releasing factor CRF_1_ receptor (Hollenstein et al., 2013), while A_2A_ receptor has been produced both in High Five (Lebon et al., 2011b) and *Sf*9 cells (Jaakola et al., 2008). Class C human metabotropic glutamate mGlu_5_ receptor was expressed in *S. frugiperda Sf*21 cells recently (Doré et al., 2014). Protein expression levels can significantly vary in different cell lines, so it is highly recommended to screen for the optimal cell line before starting large-scale production of the studied GPCR. For example, High Five cells express twice as much N-terminally truncated turkey β_1_-adrenoceptor construct compared to *Sf*9 cells (Warne et al., 2003).

The production of GPCRs in insect cells is based on infection with lytic baculovirus, a modified *Autographa californica* multiple nuclear polyhedrosis virus, which carries a GPCR gene under control of a strong polyhedrin promoter. Expression of polyhedrin is not essential in tissue culture, so its gene can be replaced with a GPCR gene. Protein expression occurs in a very late stage of infection when the lytic virus kills the host cells. This results in high levels of expression of the target protein.

There are two main approaches for preparing baculovirus with a GPCR gene. In the first approach, the engineered baculoviral DNA with a lethal deletion is cotransfected with a linearized complementary baculovirus transfer vector carrying a GPCR gene under control of polyhedrin promoter. Virus viability is restored by homologous recombination, so only viruses which carry the GPCR gene are viable (Kitts and Possee, 1993). This approach is used in e.g., BaculoGold (BD Biosciences), BestBac (Expression Systems) and Sapphire (Allele Biotechnology) baculovirus expression systems. The second approach, used in e.g., Bac-to-Bac baculovirus expression system (Invitrogen), is based on site-specific transposition of an expression cassette from a donor vector into the parent baculovirus shuttle vector (bacmid) in *Escherichia coli* DH10Bac competent cells (Luckow et al., 1993). Insertion of the expression cassette disrupts the lacZ sequence in a bacmid, so the bacterial colonies with the recombinant bacmid can be detected by blue/white selection. Insect cells are then transfected with the recombinant bacmid to produce a virus with the gene of interest. *Sf*9 or *Sf*21 cells are preferably used for either cotransfection or transfection with bacmid, because they allegedly show higher transfection efficiency and virus replication than High Five cells. In all cases, the recombinant virus is amplified in successive rounds of infection (usually two) and finally used for protein expression in the selected insect cells. It is essential to quantify virus concentration by one of a few available methods: e.g., plaque assay, end-point dilution assay, in flow cytometry analysis after immunostaining with gp64-PE antibody (Expression Systems) or ligand-biding assays to measure amounts of expressed protein. Multiplicities of infection in a range of 5–10 viral particles per insect cell are usually necessary for efficient protein expression.

As for mammalian cells, production of GPCRs in insect cells is a time-consuming procedure. It can take up to a month to produce enough baculovirus for large-scale expression. Also, growth medium is complex and quite expensive. In comparison to mammalian cells, which are grown at 37°C, insect cells are grown at 27°C. This is reflected in different membrane composition as well. Insect cell membranes are low in cholesterol, have very high phosphatidyl inositol content and no phosphatidyl serine. To make them more similar to the mammalian membranes, lipids can be added to growth media for expression of vertebrate GPCRs. Thus for expression of turkey β_1_-adrenoceptor, insect cells were grown in medium supplement with a mixture of lipids (Warne et al., 2003), while in case of human D_3_ receptor cholesterol was added to the medium 24 h after final infection (Chien et al., 2010). In some cases, the growth medium was supplemented with an antagonist that served as a pharmacological chaperone to assist receptor folding and provide additional stabilization of the mature receptor molecule (Manglik et al., 2012; Wu et al., 2012; Yin et al., 2015). Many posttranslational modifications similar to those in mammalian cells are also possible in insect cells, but there are several instances for which non-homogeneous glycosylation in insect cells resulted in immature protein. Moreover, for angiotensin AT_1_ and adenosine A_1_ receptors it was recently observed that a proportion of not properly folded receptor molecules is higher for insect cell expression in comparison to expression in stable human HEK293S(TetR) GnTI^-^ or T-Rex-293 cells (Thomas and Tate, 2014). The authors attributed this observation to impairment of secretory pathway caused by baculovirus infection and overproduction of mRNA of a targeted GPCR under control of a strong polyhedrin promoter. They also noticed that turkey β_1_-adrenoceptor is a mixture of both folded and misfolded molecules, so the success in crystallizing this receptor can be attributed to purification procedures and/or the crystallization process itself (Thomas and Tate, 2014). It is likely that the same holds true for many other GPCRs expressed in insect cells.

### Yeast

Expression in methylotrophic yeast *Pichia pastoris* has been used for production of human histamine H_1_ receptor in fusion with T4L (Shiroishi et al., 2011) as well as for human adenosine A_2A_ receptor (Yurugi-Kobayashi et al., 2009) which has been cocrystallized with an antagonist and an antibody Fab fragment (Hino et al., 2012). Different constructs of human A_2A_ receptor have also been produced for crystallographic studies in *Sf*9 and High Five insect cells (**Table 1**). *P. pastoris* is the preferred yeast organism as it gives higher functional expression levels of GPCRs (Lundstrom et al., 2006). On the other hand, *Saccharomyces cerevisiae* is more suitable for cloning and rapid screening of the protein constructs (Shiroishi et al., 2012) and it was used for both H_1_ and A_2A_ receptors to assemble the construct from PCR fragments by yeast homologous recombination. The amplified plasmids with the GPCR genes were linearized and transformed into the protease-deficient* P. pastoris* SMD1163 strain. The transformants were selected for the highest expression levels and the best one was used for large-scale production. It was shown that ligands and dimethyl sulfoxide in growth media increase the functional expression levels of GPCRs in *P. pastoris* (André et al., 2006).

In comparison to mammalian and insect cells, yeast cells grow very quickly and to higher cell densities, are easier to scale up and require relatively inexpensive media. Although they are also eukaryotic and can perform most posttranslational modifications, glycosylation patterns are different than in mammalian or insect cells. Their membranes possess higher ergosterol and much lower cholesterol content than the membranes of mammalian cells. Despite these differences, the two published GPCR structures and a large-scale study on expression of 100 GPCRs (Lundstrom et al., 2006) showed that yeast can indeed be a viable expression system for structural studies of GPCRs.

### Escherichia coli

Similar to yeast, *E. coli* provides many advantages as an expression system. It has short doubling time, can be grown to higher cell densities in inexpensive media and can easily be genetically manipulated by transformation. Although most commonly used to express soluble proteins for structural studies, prokaryotic *E. coli* cells do not contain all necessary machinery to appropriately process eukaryotic integral membrane proteins. Majority of posttranslational modifications (like glycosylation, phosphorylation, and palmitoylation) are missing from mammalian proteins produced in *E. coli*. Also, the functional folding of most GPCRs is dependent on correct formation of disulfide bonds in their extracellular region, so the reductive periplasmic environment of *E. coli* is not optimal for their functional production. In addition, lipidic composition of the bacterial inner membrane is significantly different from that of eukaryotic cells and completely lacks cholesterol, one of the main constituents of the mammalian plasma membrane.

Nevertheless, for some GPCRs it was possible to establish functional expression in the inner membrane of *E. coli*. In all these cases, a GPCR had to be fused with proteins which direct insertion into the inner bacterial membrane. The first such example is human β_2_-adrenoceptor N-terminally fused with cytoplasmic β-galactosidase (Marullo et al., 1988). MBP proved to be a very efficient N-terminal fusion partner guiding the expression into the inner membrane, as shown for a few GPCRs (Bertin et al., 1992; Grisshammer et al., 1993; Weiß and Grisshammer, 2002; Serrano-Vega et al., 2008). Even higher expression levels were obtained if, in addition to MBP as the N-terminal fusion partner, TrxA was added at the C-terminus (Tucker and Grisshammer, 1996; Furukawa and Haga, 2000; Yeliseev et al., 2007). More recently, combination of N-terminally attached Mistic and C-terminally attached TarCF gave functional expression of human CB_2_ receptor in *E. coli* (Chowdhury et al., 2012).

After some skepticism whether *E. coli* can be used as an expression system for crystallographic studies of mammalian GPCRs at all, Plückthun’s group published crystal structures of three thermostabilized variants of rat neurotensin NTS_1_ receptor (Egloff et al., 2014b). These were expressed as fusions with MBP at the N-terminus and TrxA at the C-terminus in the inner cytoplasmic membrane of *E. coli* BL21 cells and crystallized after cleaving off the fusion partners. Although the structure of rat NTS_1_ receptor fused with T4L in ICL 3 and produced in insect cells had already been known (White et al., 2012), the new NTS_1_ receptor structures revealed the amphipathic helix 8, which is absent in the older structure, as well as some differences in the ligand-binding site.

When expression of the functional membrane-inserted protein does not work, GPCRs can be expressed as inclusion bodies composed of misfolded, aggregated and almost pure protein (Lundstrom et al., 2006; Michalke et al., 2009). In such a way, higher expression levels might be reached. Namely, inclusion bodies are resistant to proteolytic digestion and serve as a convenient way to eliminate potential cell toxicity of the overexpressed protein. The main difficulty remains in getting the functional protein from the inclusion bodies. For that, they have to be dissolved and the protein folded to the native state (reviewed in Banères et al., 2011). Sometimes, GPCRs can be expressed in inclusion bodies without any fusion partner (Bane et al., 2007; Michalke et al., 2010). There are also some fusion proteins that target GPCRs into inclusion bodies: GST, TrxA and the fragment of human α_5_ integrin are the most efficient ones (Kiefer et al., 1996; Michalke et al., 2009; Arcemisbéhère et al., 2010).

Expression of GPCRs as inclusion bodies in *E. coli* and subsequent refolding can indeed be used in their structural studies, as confirmed by the NMR structure of human chemokine CXCR1 (Park et al., 2012b). The isotopically labeled GST fusion of CXCR1 was expressed as inclusion bodies in *E. coli* (BL21). The fusion partner was cleaved off and the receptor molecule reconstituted into proteoliposomes to measure solid-state NMR spectra.

### Cell-Free Expression

Cell-free expression becomes used more often for large-scale production of integral membrane proteins with yields up to ∼ 1 mg per mL of reaction mixture (reviewed in Junge et al., 2011; Rajesh et al., 2011; Bernhard and Tozawa, 2013). *E. coli* extract (Schwarz et al., 2007) is most common, although more complicated and expensive eukaryotic cell extracts from wheat germ embryos or rabbit reticulocytes have also been used for GPCR production (Robelek et al., 2007; Kaiser et al., 2008; Katzen et al., 2008). Cell-free expression based on a standard wheat germ extract has decoupled translation, which means that mRNA with the gene of interest has to be provided for translation to occur. For all cell-free syntheses of GPCRs, continuous exchange configuration has been applied.

**FIGURE 2:**
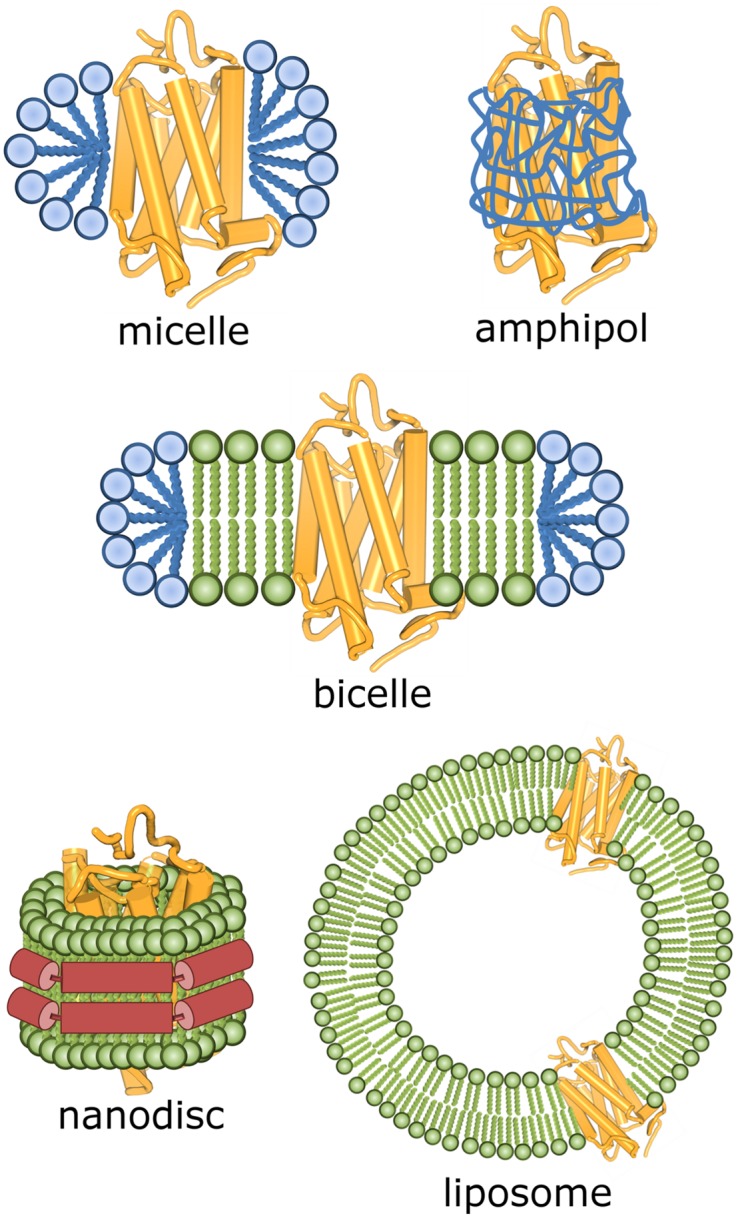
**Schematic representation of some possible environments for GPCR molecules after their extraction from biological membranes**.

Membrane proteins in cell-free settings can be expressed either as insoluble precipitates or directly in a soluble form if proper detergents or membrane mimetics are present. Cell-free expression as precipitate and subsequent post-translational solubilization in a series of detergents did not yield functional receptors (Klammt et al., 2005, 2007b). On the other hand, when human ET_B_ receptor was expressed as a precipitate, solubilized in detergent LMPG and reconstituted into lipids, partial folding of the receptor molecule was observed (Proverbio et al., 2013). Cell-free expression of ET_B_ receptor in a presence of lipids also resulted in the functional receptor (Proverbio et al., 2013). In presence of mild detergents, like steroid detergent digitonin or the long-chain polyoxyethylene derivatives (Brij), cell-free synthesis gave functional proteins after their insertion into liposomes (**Figure 2**) for a number of GPCRs (Ishihara et al., 2005; Klammt et al., 2007a,b; Kaiser et al., 2008; Corin et al., 2011a; Proverbio et al., 2013; Wang et al., 2013c,d). In addition, newly developed peptide surfactants (Corin et al., 2011b; Wang et al., 2011), a fructose-based NV10 polymer (NVoy; Klammt et al., 2011), nanodiscs (Katzen et al., 2008; Yang et al., 2011; Proverbio et al., 2013) and a solid-supported lipid membrane that mimics a biological membrane (Robelek et al., 2007) have been used instead of detergents for cell-free production of several functional GPCRs.

Expression efficiency in a cell-free system sometimes depends on an N-terminal tag used, with TrxA and some smaller tags, like T7-tag, being most successful (Ishihara et al., 2005; Haberstock et al., 2012; Lyukmanova et al., 2012). This is explained by different secondary structure of the corresponding mRNA affecting the translation efficiency.

Until now, there are no structural reports of GPCRs produced in a cell-free system. The NMR structure of the bacterial proteorhodopsin (Reckel et al., 2011) and the X-ray structure of eukaryotic *Acetabularia* rhodopsin II from marine alga (Wada et al., 2011), the two 7TM proteins produced by cell-free syntheses, suggest that this protein production method might gain a more prominent role in future structural studies of GPCRs.

### Other Expression Systems

Some prokaryotic organisms have been tried for production of GPCRs, including archaeon *Haloferax volcanii* (Patenge and Soppa, 1999) and photosynthetic bacterium *Rhodobacter sphaeroides* (Roy et al., 2008), but none of them resulted in successful structural studies. Eyes of transgenic fruit flies (*Drosophila melanogaster*; Eroglu et al., 2002; Panneels and Sinning, 2010) as well as fat body and silk glands of transgenic silkworms (*Bombyx mori*; Tateno et al., 2009) gave expression levels of the studied GPCRs similar or even higher than in *Sf*9 cells. Furthermore, ROS membranes of transgenic *Xenopus laevis* tadpoles (Zhang et al., 2005) and transgenic mice (Li et al., 2007) gave homogeneously glycosylated and functional GPCRs. Rabbit chemokine receptor CXCR1 was expressed in mouse liver infected with the adenovirus (Sarmiento et al., 2009). The authors estimated that 20 mice livers would be enough to produce 1 mg of the receptor. More recently, milligram quantities of several functional GPCRs were expressed in muscles and neurons of a worm *Caenorhabditis elegans* (Salom et al., 2012). Taking into account how difficult and expensive it is to create the transgenic animals, this is definitely not the first method of choice to produce GPCRs for structural studies.

## Solubilization

### Cell Disruption and Membrane Preparation

To perform structural studies, GPCRs have to be extracted from the biological membranes and purified. Mammalian and insect cells are easier to break and usually hypotonic buffer in combination with one freeze-thaw cycle is enough to completely break the cells. In contrast, yeast and bacterial cells possess cell walls, so usually mechanical force, like shaking with glass beads (Yurugi-Kobayashi et al., 2009; Shiroishi et al., 2011) or sonication (Egloff et al., 2014b), has to be used for complete cell breakage. GPCRs are notoriously thermally unstable when outside of their native membrane environment, hence it is important to take all necessary care not to overheat or mechanically damage the sample during the mechanical cellular disruption. Also, protease inhibitors should always be included to prevent proteolytic digestion of flexible GPCR molecules.

After cellular disruption, the usual next step is membrane preparation. This includes ultracentrifugation and extensive washing with a high osmotic buffer containing up to 1.0 M NaCl, which removes practically all cytoplasmic proteins, or – as in case of bovine and squid rhodopsin isolated from natural sources – sucrose density gradient preparation (Kito et al., 1982; Okada et al., 1998). Although membrane preparation enriches a crude sample with the overexpressed membrane protein, in several instances GPCRs have been isolated for structural studies directly from mammalian (Standfuss et al., 2007, 2011; Deupi et al., 2012; Singhal et al., 2013), insect (Kruse et al., 2012; Manglik et al., 2012; Zhang et al., 2012; Zou et al., 2012) or *E. coli* (Egloff et al., 2014b) cells without intermediate preparation of membranes.

### Solubilization – a Role of Detergents in Structural Studies

Before any further purification, GPCRs have to be solubilized in detergents. If concentration of detergent molecules (monomers) in water solution is higher than the CMC, detergent molecules associate into aggregates – detergent micelles – with the hydrophilic heads at the surface of a micelle and hydrophobic tails in its interior. The CMC is specific for each detergent and also depends on a variety of factors, like ionic strength, pH and temperature. For solubilization it is important that the detergent concentration is high enough to disrupt the membrane and form mixed micelles containing both membrane proteins and lipids. In such protein–detergent complexes, protein hydrophobic surfaces are screened with detergent hydrophobic tails, while the hydrophilic regions are exposed to the solvent (**Figure 2**). The protein–detergent complex can be imagined as protein molecule surrounded by a belt of detergent molecules and co-solubilized membrane lipids. It is very important that the chosen detergent is efficient enough in solubilizing the protein from the membrane, but still mild enough not to deteriorate the protein native structure and functionality. As a rule of thumb, charged detergents with smaller hydrophilic heads and shorter hydrophobic tails are harsher than non-ionic detergents with larger hydrophilic heads and longer hydrophobic tails. It is impossible to predict how a particular detergent interacts with a GPCR, so the most suitable detergent for solubilization and further purification has to be found experimentally.

**FIGURE 3:**
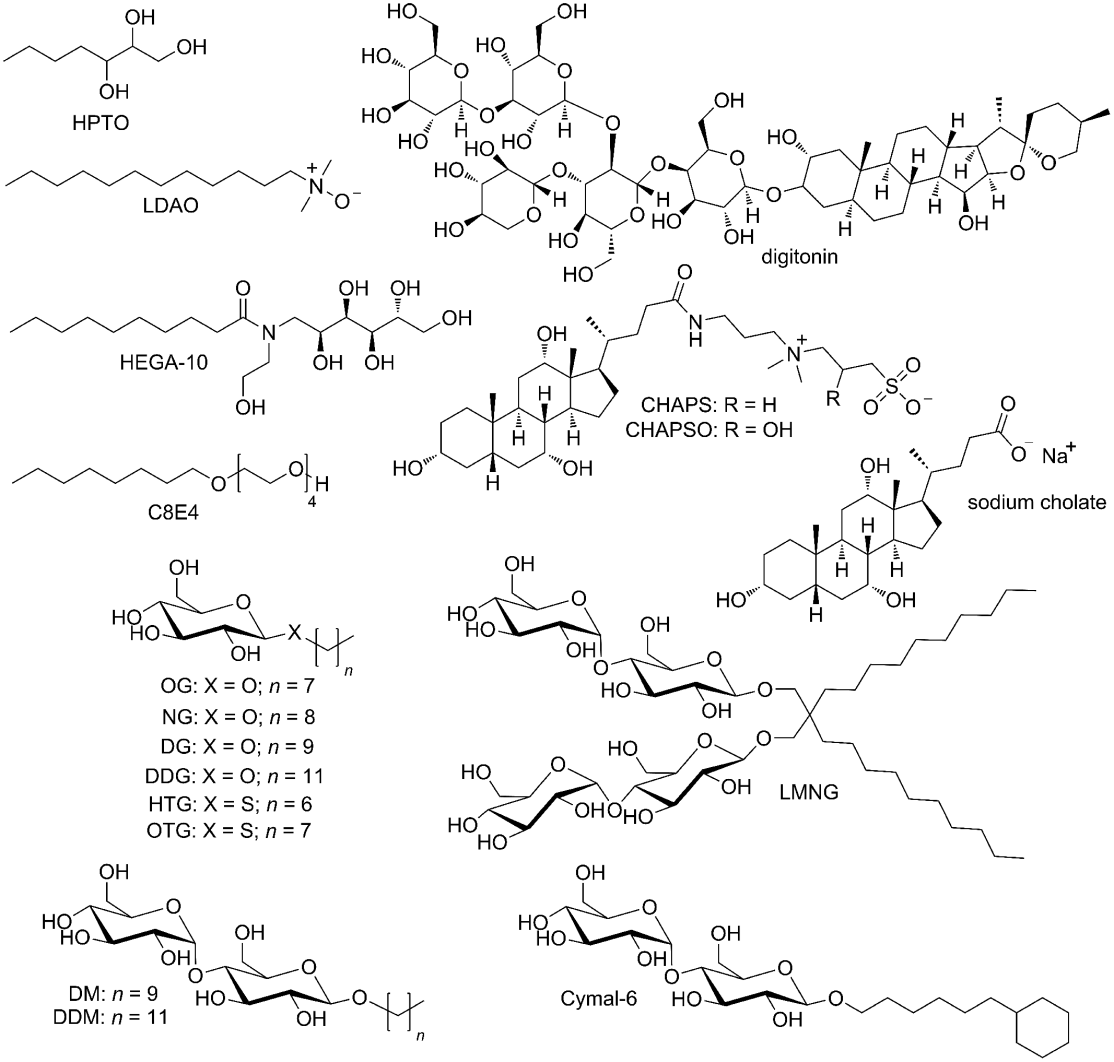
**Detergents used in solubilization and purification of rhodopsin and other GPCRs for crystallographic studies**.

A rather robust GPCR, rhodopsin, could be solubilized from bovine ROS membranes without losing its structural integrity in a variety of detergents (**Figure 3**): a mixture of HPTO and NG (Okada et al., 1998, 2000; Salom et al., 2006a), OG (Park et al., 2008, 2013; Scheerer et al., 2008; Choe et al., 2011), short-chain HTG (Okada et al., 2004), zwitter-ionic LDAO (Edwards et al., 2004) and a mixture of OG and mild DDM (Choe et al., 2011). Bovine rhodopsin mutants produced in mammalian cells were solubilized in DM (Standfuss et al., 2007) or DDM (Standfuss et al., 2011; Deupi et al., 2012; Singhal et al., 2013).

For crystallographic studies, non-rhodopsin GPCRs are most often solubilized in DDM (concentration: 0.5–1.0%, w/v), although other detergents are employed as well, including DM (1.5–2%; Warne et al., 2009; Hollenstein et al., 2013), a newly developed lauryl-maltose-neopentyl glycol (LMNG or MNG-3, 1%; Chae et al., 2010; White et al., 2012) as well as mixtures of: digitonin (1%) and sodium cholate (0.35%; Haga et al., 2012), DDM (1%) and sodium cholate (0.2%; Zhang et al., 2012; Yin et al., 2015), LMNG (1.0%) and sodium cholate (0.3%; Granier et al., 2012), DM (1.5%) and zwitter-ionic CHAPS (0.8 %; Egloff et al., 2014b), and finally, DDM (0.5%) and CHAPS (0.3%; Manglik et al., 2012).

A cholesterol derivative CHS additionally stabilizes GPCRs and maintains their activity in detergent solutions, as first observed for human A_2A_ receptor (Weiß and Grisshammer, 2002). It is usually added to solubilization buffers in concentration 0.1–0.3% (w/v) and kept further in subsequent purification buffers in 10–30 times smaller concentration. Hanson et al. (2008) established the cholesterol consensus binding motif which is present in 44% of human class A GPCRs (Hanson et al., 2008). Indeed, specific cholesterol binding sites have been indentified in crystal structures of human β_2_-adrenoceptor (Cherezov et al., 2007; Hanson et al., 2008; Wacker et al., 2010; Rosenbaum et al., 2011), turkey β_1_-adrenoceptor (Warne et al., 2011; Christopher et al., 2013), human A_2A_ receptor (Liu et al., 2012b), human 5-HT_2B_ receptor (Liu et al., 2013; Wacker et al., 2013) as well as human P2Y_12_ receptor (Zhang et al., 2014b).

G protein-coupled receptors are less stable in high concentrations of detergents used for solubilization, so detergent concentrations are gradually reduced in further purification steps. It is important for detergent concentration to stay above the CMC value; therefore, concentrations corresponding to 2–3 CMC are usually applied. Detergent concentrations lower than CMC might cause dissociation of protein–detergent complex and subsequent protein aggregation. Sometimes a detergent which is very efficient in GPCR solubilization might not be the optimal one for structural studies. If crystallization of GPCR is pursued *in surfo*, i.e., from protein–detergent complex in water solutions, detergents which form smaller micelles are preferred. Their molecules screen less of GPCR hydrophilic surfaces, produce smaller protein–detergent complexes (Privé, 2007) and thus favor a formation of crystal contacts between GPCR molecules. As a counter-effect, these detergents are usually more destabilizing (harsher), therefore it is crucial that GPCR monodispersity and functionality are carefully evaluated before trying crystallization. Smaller protein–detergent complexes, having faster tumbling, are also preferred for solution-state NMR spectroscopy.

Detergents successfully used for *in surfo* crystallization of bovine and squid rhodopsins include a mixture of HPTO and NG (Okada et al., 2000), HTG (Okada et al., 2004), LDAO partially exchanged with C8E4 (Edwards et al., 2004), C8E4 (Standfuss et al., 2007), NG alone (Salom et al., 2006a), OG alone (Murakami and Kouyama, 2008; Park et al., 2008; Deupi et al., 2012) or in a mixture with DDM (Choe et al., 2011), and a mixture of DDM and LDAO (Shimamura et al., 2008; **Figure 3**). Several other GPCRs have also been crystallized *in surfo*. Diffraction-quality crystals of the thermostabilized turkey β_1_-adrenoceptor were obtained in OTG (Warne et al., 2008, 2009), HEGA-10 (Moukhametzianov et al., 2011; Warne et al., 2011) and even in DDM supplemented with a mixture of four lipids (Huang et al., 2013). The thermostabilized variants of human A_2A_ receptor were crystallized *in surfo* from NG with addition of 6-cyclohexyl-1-hexyl-β-D-maltopyranoside (Cymal-6; Doré et al., 2011) and OTG with CHS (Lebon et al., 2011a), while OTG only, without additional lipids, was enough for *in surfo* co-crystallization of the non-stabilized receptor with Fab fragment (Hino et al., 2012). Egloff et al. (2014b) used a mixture of NG, DG, DDG, and CHS in vapor diffusion experiments to prepare crystals of the thermostabilized variants of rat NTS_1_ receptor expressed in *E. coli*.

In contrast to *in surfo* crystallization, less consideration for detergent needs to be given for *in meso* crystallization methods. These are performed in membrane-mimetic environment, either in bicelles (Ujwal and Bowie, 2011) or in lipidic mesophases (Caffrey, 2015). Bicelles are disk-like lipidic bilayer patches which are surrounded and stabilized by amphiphile (usually detergent) molecules (**Figure 2**). They provide almost native environment to a membrane protein, but their relatively large size is a disadvantage for their use in crystallization or solution-state NMR spectroscopy. Nevertheless, the structure of human β_2_-adrenoceptor in complex with a Fab fragment was obtained in vapor diffusion crystallization from bicelles made from a mixture of DMPC and CHAPSO in DDM (Rasmussen et al., 2007).

**FIGURE 4:**
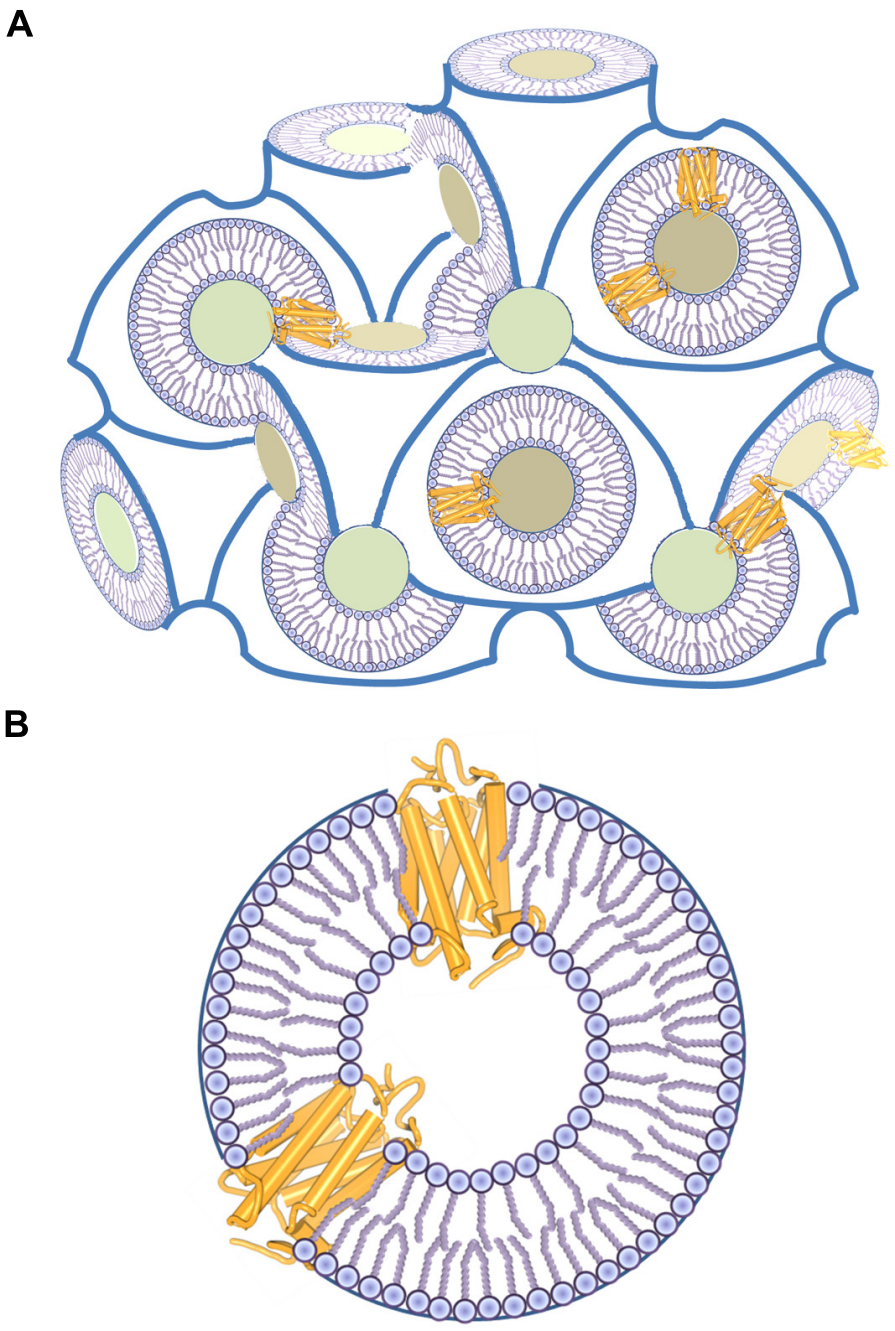
**Structure of a LCP. (A)** LCP consists of water channels (shown as colored cross-sections) and a continuous 3D lipid bilayer that allows free diffusion of the reconstituted membrane protein molecules. **(B)** A detailed view of GPCR molecules in a LCP bilayer.

The most used systems for crystallization of GPCRs are lipidic mesophases (reviewed in Yin et al., 2014; Caffrey, 2015). LCPs are bicontinuous liquid crystals composed of a single, curved lipidic bilayer that separates two continuous, non-contacting channels filled with water medium (**Figure 4**). As a lipidic component different monoacylglycerols are used and among them monoolein (MAG9.9) is the most common one (Caffrey, 2015). LCP forms by mixing e.g., monoolein with water solution containing a solubilized membrane protein in a 3:2 weight ratio at 20°C. In this process, a membrane protein gets incorporated into the stabilizing and native-like environment of a lipidic bilayer. In certain conditions LCP transforms into lipidic sponge phase, which essentially preserves its bicontinuous structure, but it is a true liquid and lacks the liquid-crystal properties. If the right crystallization conditions are encountered, both LCP and lipidic sponge phase support crystal nucleation and growth. The most GPCR crystal structures were obtained from crystallization in a lipidic mesophase. In almost all cases, a mixture of monoolein and cholesterol in 9:1 weight ratio was used, because – as already discussed – cholesterol makes GPCRs more stable, less conformationally flexible and thus increases probability of their crystallization. A notable exception is the β_2_-adrenoceptor–G_s_ protein complex (Rasmussen et al., 2011b), for which a mixture of MAG7.7 and cholesterol in a weight ratio of 9:1 was used instead of a monoolein–cholesterol mixture. Rationale for this is the fact that MAG7.7 forms LCP with water channels that are large enough to accommodate a relatively large heterotrimeric G protein in the macromolecular complex (**Figure 1**).

Because a membrane protein is incorporated in a lipidic bilayer (**Figure 4**) and mechanisms of protein crystallogenesis are different than for *in surfo* crystallization, the nature of detergent or other surfactant used to keep GPCR soluble in water plays a less important role for this kind of *in meso* crystallization. DDM in a mixture with CHS is the most common detergent used in purification of GPCRs for LCP crystallization. There are also several instances where LMNG was used instead, both alone (Rasmussen et al., 2011a,b; Rosenbaum et al., 2011; Haga et al., 2012; Yin et al., 2015) or mixed with CHS (Granier et al., 2012; Kruse et al., 2012; Manglik et al., 2012; White et al., 2012). Actually, LMNG might even be a better choice for LCP crystallization than DDM. It can provide higher stability to solubilized GPCR molecules and has lower CMC (Chae et al., 2010). Namely, higher detergent concentration not only destabilizes native protein conformation, but it also prevents formation of LCP (Ai and Caffrey, 2000; Misquitta and Caffrey, 2003). One should always be careful not to exceed the maximal tolerable detergent concentration in the last protein concentrating step, in which empty detergent micelles might be concentrated together with protein–detergent complexes.

After solubilization, detergents can be exchanged for other surfactants that are not capable to extract a membrane protein from a membrane (**Figure 2**). Amphipathic polymers, amphipols, proved to be very efficient in stabilizing and even refolding of GPCRs expressed as inclusion bodies (Banères et al., 2011; Popot et al., 2011; Mary et al., 2014). Some types of amphipols are suited for solution-state NMR studies of membrane proteins (Planchard et al., 2014) and there is also a recent report of using amphipols for crystallization of a 7TM-protein bacteriorhodopsin in LCP (Polovinkin et al., 2014). In the future, amphipols might get more pronounced role in GPCR structural studies. We have already mentioned bicelles that can be used both for crystallization (Rasmussen et al., 2007) and for NMR studies (Werner et al., 2007; Yoshiura et al., 2010). GPCRs incorporated into liposomes can be studied by solid-state NMR (e.g., CXCR1; Park et al., 2012b). Nanodiscs are lipid bilayers stabilized by the surrounding scaffold protein. They provide stabilizing environment for both biophysical (Bayburt et al., 2007; Bokoch et al., 2010) and NMR studies of membrane proteins (Raschle et al., 2009; Hagn et al., 2013).

Recently, a very interesting approach was demonstrated, which avoids use of detergents or any other surfactants in GPCR studies. Namely, Perez-Aguilar et al. (2013) computationally designed a water-soluble form of human μ opioid receptor and managed to overexpress this engineered soluble protein with a yield of ∼20 mg per L of a shake flask *E. coli* culture (Perez-Aguilar et al., 2013). For this, they had to mutate 53 exterior residues in the transmembrane region of the receptor mainly to the hydrophilic ones. The resulting receptor variant was not only water soluble, but it showed α-helical secondary structure and affinity for the antagonist very similar to the native receptor.

## Purification

Due to their abundance in the natural sources, bovine and squid rhodopsins could be crystallized immediately after extraction with detergents from the fractionated membranes without any further purification (Okada et al., 2000; Murakami and Kouyama, 2008). This is not possible for heterologously expressed GPCRs. After solubilization they always need some robust purification protocol prior to structural studies.

In many cases, thiol groups of free cysteine residues in receptor molecules are blocked with iodoacetamide to prevent protein aggregation by intermolecular disulfide-bond formation. High-affinity orthosteric and allosteric ligands – especially antagonists and inverse agonists – generally increase receptor stability, so GPCRs are almost always solubilized and purified in their presence. Furthermore, covalent agonists have been developed for β_2_-adrenoceptor and used in crystallographic studies (Rosenbaum et al., 2011; Weichert et al., 2014). They covalently and irreversibly bind to an engineered cysteine residue in an orthosteric binding site and, together with a G-protein-mimetic nanobody, stabilize the receptor in an active conformation. We also remind that the retinal isomers are natural covalent ligands to (rhod)opsin.

Detergents are exchanged stepwise when protein is immobilized on an affinity chromatographic column or during size exclusion chromatography. Special care should be taken to minimize buffer volumes used in washing and detergent exchange as this might wash away co-purified lipids that additionally stabilize the solubilized receptor.

### Affinity Chromatography

Immobilized metal-ion affinity chromatography is commonly used as the first chromatographic step in GPCR purification. For that, polyhistine-tag (His-tag) is introduced at the N- or C-terminal part of the expression construct. Deca histidine-tag is the most common one, although octa (e.g., Shiroishi et al., 2011; Granier et al., 2012; Manglik et al., 2012) and hexa (e.g., Warne et al., 2003; Kruse et al., 2012; Park et al., 2012b; Huang et al., 2013) histidine-tags have also been used successfully. Both Ni^2+^–NTA and Co^2+^–CMA matrices showed to be efficient in IMAC of different GPCRs. They both tolerate detergents in amounts used for solubilization, but Co^2+^–CMA (TALON resin) shows less non-specific binding and higher elution purity (Bornhorst and Falke, 2000), so it is preferred for purification of GPCRs with lower expression levels. In addition, batch mode increases GPCR binding to IMAC resins.

There are only several instances of GPCRs purified for crystallization for which IMAC was not the first chromatographic step. ConA affinity chromatography, which can separate glycosylated from non-glycosylated proteins, have been used in Schertler’s group for the enrichment of bovine rhodopsin isolated from ROS membranes (Edwards et al., 2004) as well as for the squid rhodopsin purification in Ishiguro’s group (Shimamura et al., 2008). In case of human A_2A_ receptor expressed in *P. pastoris*, ConA affinity chromatography was used as the second chromatographic step to remove contaminating glycosylated proteins (Hino et al., 2012).

Standfuss et al. (2007, 2011), Deupi et al. (2012) and Singhal et al. (2013) used Rho1D4-antibody resin for binding of bovine rhodopsin mutants after solubilization from the mammalian cells. The monoclonal Rho1D4-antibody is highly specific for the nine C-terminal rhodopsin residues (TETSQVAPA), so this C-terminal amino acid sequence (1D4- or Rho9-tag) can also be used as an affinity-tag for purification of integral membrane proteins (Wong et al., 2009). An engineered N-terminal FLAG-tag (DYKDDDDA or DYKDDDDK) was employed in isolation of β_2_-adrenoceptor (Kobilka, 1995) by FLAG M1 antibody affinity chromatography for a number of crystal structures, either in the two chromatographic steps (Rasmussen et al., 2007, 2011a,b; Zou et al., 2012) or only in the initial one (Bokoch et al., 2010; Rosenbaum et al., 2011). Having two different affinity tags (e.g., FLAG- and His-tags) at two ends of a GPCR molecule is in general an advantage. By using the two different affinity chromatographic methods in purification, one secures that the purified construct is intact at both N- and C-terminus. Along these lines, a FLAG M1 antibody affinity resin was applied in the second purification step after initial Ni-NTA IMAC isolation of several GPCRs (Granier et al., 2012; Kruse et al., 2012; Manglik et al., 2012; Zhang et al., 2012; Yin et al., 2015).

If there is an available resin with immobilized ligand for the GPCR of interest, ligand-affinity chromatography can be applied as an important purification step. Moreover, it can separate properly folded protein with preserved ligand-binding properties from misfolded and unfolded GPCR molecules. One of the oldest ligand-affinity resins is alprenolol-resin developed for purification of adrenoceptors. It has been used in purification for crystallographic studies of human β_2_-adrenoceptor (Rasmussen et al., 2007, 2011b) and turkey β_1_-adrenoceptor (Warne et al., 2008). Furthermore, ligand-affinity chromatography has been applied as the first chromatographic step in purification of M_2_ receptor (aminobenztropine-resin; Haga et al., 2012) and NTS_1_ receptor expressed in *E. coli* (protease cleavable pD-NT-resin; Egloff et al., 2014a,b).

### Ion-Exchange Chromatography

Although not as general as purification methods based on the affinity-tags, ion-exchange chromatography was used in purification of several GPCRs. Bovine rhodopsin isolated from ROS (Edwards et al., 2004) as well as its thermostabilized mutant expressed in mammalian cells (Standfuss et al., 2007) were both purified on an anion-exchange MonoQ resin. Shimamura et al. (2008) used inverse purification on anion-exchange DEAE-cellulose for purification of squid rhodopsin. Because methylation destroyed the receptors antigenicity toward FLAG-antibody, the methylated human β_2_-adrenoceptor had to be purified on the Q sepharose anion exchange resin (Bokoch et al., 2010). Cation exchange SP sepharose was used in the last purification step before crystallization of rat NTS_1_ receptor expressed in *E. coli* (Egloff et al., 2014b). Similarly, the last step before crystallization of human M_2_ receptor was binding to a hydroxyapatite column and exchange of detergents (Haga et al., 2012).

### Size-Exclusion Chromatography

Even though SEC is often considered a polishing purification step before structural studies, it has not been used so much in GPCR purification as it might be expected. The majority of GPCR structures have been determined from crystals formed in LCP. *In meso* LCP crystallization is more robust in comparison to the *in surfo* methods and tolerates higher amounts of protein impurities and aggregates (Kors et al., 2009). SEC has been used as a final step before *in surfo* crystallization of the bovine rhodopsin mutants (Standfuss et al., 2011; Deupi et al., 2012; Singhal et al., 2013) as well as human A_2A_ receptor in complex with agonist (Lebon et al., 2011b). Purification of macromolecular complexes involving human β_2_-adrenoceptor (Rasmussen et al., 2007, 2011b; Ring et al., 2013), A_2A_ receptor (Hino et al., 2012) or M_2_ receptor (Kruse et al., 2013) were also finalized with SEC. In a few other cases, SEC was used primarily to purify the GPCR sample after proteolytic cleavage (Granier et al., 2012; Kruse et al., 2012; Manglik et al., 2012; Zhang et al., 2012; Zou et al., 2012) or deglycosylation (Yin et al., 2015). In preparation of CRF_1_ receptor for the LCP crystallization (Hollenstein et al., 2013) as well as CXCR1 for NMR studies (Park et al., 2012b), there was only one IMAC purification step before the final SEC purification.

### Proteolytic Cleavage

Affinity tags are very useful in purification, but they can interfere with the structural studies. These very flexible regions might prevent crystallogenesis or produce intense and overlapping NMR signals. Also, the native GPRC N- and C-termini might be important for functional expression, but their flexibility might have the same negative effects as affinity tags. Hence, in purification procedures of many GPCRs, these flexible parts are removed by action of proteases (reviewed in Waugh, 2011).

Vergis and Wiener (2011) made an exhaustive systematic study of detergent sensitivities of the most common proteases. They found that thrombin was the only protease with practically intact activity in all detergents tested. Indeed, thrombin has been used for removal of GST from the CXCR1 construct expressed in *E. coli* for NMR studies (Park et al., 2012b). Although very active in a variety of detergents, thrombin is not an optimal protease for cleavage of recombinant GPCR constructs. It is purified from bovine plasma, so it lacks affinity tag which could provide easier removal after cleavage. In addition, it can show unpredictable non-specific cleavage in certain conditions (Jenny et al., 2003). On contrary, TEV protease and HRV 3C protease are highly specific, both can easily be produced “in-house” with different affinity tags and show reasonable activities in detergents commonly used in structural studies (Vergis and Wiener, 2011). Indeed, TEV protease and, to a lesser extent, HRV 3C protease (also in its commercial form as PreScission protease) have been used for removal of affinity tags (His- or FLAG-tag in almost all GPCR constructs used for crystallographic studies), fusion partners (like GFP, in human H1 receptor; Shimamura et al., 2011) or flexible N-terminal region important for GPCR expression, but interfering with its crystallization (Rasmussen et al., 2007; Rosenbaum et al., 2011; Granier et al., 2012; Kruse et al., 2012; Manglik et al., 2012; Zhang et al., 2012). Carboxypeptidase A was used to remove octa histidine-tag at the C-terminus of mouse opioid μ and δ receptors (Granier et al., 2012; Manglik et al., 2012), while the very long and flexible C-terminal proline repeats of squid rhodopsin have been cleaved off by endoproteinase Glu-C from *Staphylococcus aureus* V8 (V8 protease) before extracting the rhodopsin from the rhabdomic microvillar membranes (Murakami and Kouyama, 2008, 2011; Shimamura et al., 2008).

### Deglycosylation

As already discussed, *N*-linked glycosylation might be very important for proper folding and functionality of GPCRs. On the other hand, glycan heterogeneity and flexibility can prevent formation of ordered GPCR crystals. If certain glycosylation sites are not crucial for GPCR folding, they can be removed from the protein construct by point mutations or truncations. Otherwise, if there is a designed protease cleavage site, the glycosylated flexible N-terminal part of a GPCR molecule can be cleaved off by the protease (Rasmussen et al., 2007; Rosenbaum et al., 2011; Granier et al., 2012; Kruse et al., 2012; Manglik et al., 2012). In most other cases, GPCRs expressed in insect cells were deglycosylated by peptide-*N*-glucosidase F (PNGase F), an amidase that cleaves between an asparagine side chain and an *N*-acetylglucosamine moiety bound directly to it. It should be noted that the glycosylated asparagine is modified to aspartate after deglycosylation with PNGase F.

Although deglycosylation is a generally preferred way of preparing GPCR samples for crystallization, in some instances high-quality crystals of the glycosylated GPCRs were obtained, including bovine and squid rhodopsins, S1P_1_ receptor (Hanson et al., 2012), SMO (Wang et al., 2014), mGlu_5_ receptor (Doré et al., 2014) and the two fusion constructs of A_2A_ receptor (Xu et al., 2011b; Liu et al., 2012b). Moreover, the glycan moieties were identified in the published crystal structures of bovine rhodopsin, S1P_1_ receptor and SMO. Glycans can be involved in crystal contacts, so uniform glycosylation might even promote formation of higher-quality crystals. This was demonstrated for the thermostabilized bovine rhodopsin for which removing of all glycosylation sites resulted in crystals of lower diffraction quality (Standfuss et al., 2007).

## Protein Engineering for Structural Studies

Except for rhodopsin isolated from bovine ROS membranes, all other GPCRs had to be modified for successful crystallization (**Figure 1**). These modifications include: (i) thermostabilizing and detergent-stabilizing point mutations, (ii) point mutations that increase expression levels, (iii) mutated glycosylation sites, (iv) truncations and deletions of flexible parts as well as (v) insertion of a water soluble fusion partner. It is not easy to predict an effect of each modification, so the optimal construct has to be found experimentally.

### Truncations and Deletions

Flexible parts in a GPCR molecule impair crystallization. Fortunately, they can be conveniently predicted (Yang et al., 2005; Ishida and Kinoshita, 2008). Class B, C, and F GPCRs possess large N-terminal extracellular domains that were omitted from the crystallization constructs. Significant portions of unstructured extracellular and/or intracellular tails were also deleted in the majority of crystallized GPCRs. Apart from bovine rhodopsin, only four GPCRs were crystallized with intact both N- and C-termini (Chien et al., 2010; Haga et al., 2012; Kruse et al., 2013; Srivastava et al., 2014; Zhang et al., 2014a). ICL 3 of class A GPCRs is usually unstructured. Therefore, it was shortened and/or substituted with a fusion protein, with notable exceptions of bovine and squid rhodopsins, β_2_-adrenoceptor cocrystallized either with Fab fragment (Rasmussen et al., 2007) or with G_s_ protein and nanobody (Rasmussen et al., 2011b), A_2A_ receptor crystallized either with Fab fragment (Hino et al., 2012) or as thermostabilized variants (Doré et al., 2011; Lebon et al., 2011b) and, finally, the N-terminal fusions of human NOP and δ receptors (Thompson et al., 2012; Fenalti et al., 2014). It should be kept in mind that significant modifications of an ICL or C-terminal truncation prevent coupling with intracellular effector proteins in most cases.

### Thermostabilizing Point Mutations

Stability of detergent-solubilized GPCRs remains a main challenge in their structural studies. If human receptor is not stable enough, one can screen for more stable orthologs. This is one of the reasons why protein from turkey erythrocytes was chosen for studies of β_1_-adrenoceptor. Similarly, GPCRs from rat and mouse were used instead of the human orthologs (**Table 1**). High-affinity ligands and/or antibodies, either nanobodies (Rasmussen et al., 2011a; Kruse et al., 2013; Ring et al., 2013; Weichert et al., 2014) or Fab fragments (Rasmussen et al., 2007; Bokoch et al., 2010; Hino et al., 2012), can provide stabilization for structural studies and lock the receptor in a particular conformation. In addition, a heterotrimeric G_s_ protein together with nanobody (Rasmussen et al., 2011b) as well as short polypeptide fragments derived from a G_t_α subunit (Scheerer et al., 2008; Choe et al., 2011; Standfuss et al., 2011; Deupi et al., 2012; Park et al., 2013; Singhal et al., 2013) or arrestins (Szczepek et al., 2014) might have the same effect. Another way (or necessity) is stabilizing a GPCR by protein engineering.

The first successful thermostabilizing mutation was a rationally engineered disulfide bridge in a molecule of bovine rhodopsin (Xie et al., 2003) resulting in crystal structures of several rhodopsin mutants (Standfuss et al., 2007, 2011; Deupi et al., 2012; Singhal et al., 2013). Based on the rhodopsin structure, Stevens’ group designed a stabilizing E122^3.41^W mutation [Ballesteros–Weinstein numbering (Ballesteros and Weinstein, 1995) used in superscript] in human β_2_-adrenoceptor (Roth et al., 2008). By stabilizing an interface between transmembrane helices 3, 4, and 5, this mutation was not only useful in obtaining new crystal structures of β_2_-adrenoceptor complexed with several inverse agonists and antagonists (Hanson et al., 2008; Wacker et al., 2010), but it was also successfully transferred to some other class A GPCRs enabling structure determination of CXCR4 (Wu et al., 2010), D_3_ (Chien et al., 2010), 5-HT_1B_ (Wang et al., 2013a) and 5-HT_2B_ (Wacker et al., 2013) receptors. Another successful rational design examples are introduction of a salt bridge into turkey β_1_-adrenoceptor, inspired by the structure of thermally more stable human β_2_-adrenoceptor and included into the ultra-thermostable β_1_-adrenoceptor variant (Miller and Tate, 2011), as well as introduction of a salt bridge and three additional stabilizing mutations, partially based on the CXCR4 structures, into CCR5 (Tan et al., 2013).

A very successful approach for increasing thermostability of GPCRs was a systematic alanine scanning mutagenesis where each amino acid residue was mutated to alanine (or to leucine, if it had already been alanine). It was applied for turkey β_1_-adrenoceptor (Serrano-Vega et al., 2008; Warne et al., 2009), human A_2A_ receptor stabilized both in the antagonist (Magnani et al., 2008; Doré et al., 2011) and agonist (Lebon et al., 2011a,b) binding conformations, NTS_1_ receptor (Shibata et al., 2009; White et al., 2012), CRF_1_ receptor (Hollenstein et al., 2013) and, most recently, for mGlu_5_ (Doré et al., 2014) and FFA1 receptor (Hirozane et al., 2014; Srivastava et al., 2014). Following expression, either in *E. coli* or in transiently transfected HEK293T cells, each mutant was solubilized in detergent solution and tested for thermostability by a radioligand binding assay. The only exception to this approach is FFA1 receptor (Hirozane et al., 2014). Mutants of FFA1 receptor were expressed in human FreeStyle293 (Life Technologies) cells following transient cotransfection with mammalian virus-like particles and the thermostabilizing mutations were identified in a binding assay based on SEC coupled with liquid chromatography–mass spectroscopy (SEC/LC-MS).

The most stabilizing mutations detected in alanine scanning mutagenesis are further combined in several additional screening rounds by subsequently adding each mutation. Only mutants giving additive stabilization are selected for further rounds. The resulted mutants are not only thermostabilized, but are also locked in a specific conformation determined by a nature of ligand used. Subsequent leucine scanning mutagenesis identified some additional stabilizing mutations in turkey β_1_-adrenoceptor (Miller and Tate, 2011). The stabilizing point mutation found for turkey β_1_-adrenoceptor were successfully transferred into highly similar human β_1_- and β_2_-adrenoceptors (Serrano-Vega and Tate, 2009). This shows that thermostabilizing mutations found in one GPCR can be transferred into another one if sequence similarity between the two receptors is high enough. For more distant GPCRs that does not have to be true.

Directed evolution is an alternative approach for finding GPCR variants with high functional expression and significantly improved thermal and detergent stability (Sarkar et al., 2008; Dodevski and Plückthun, 2011; Schlinkmann et al., 2012a,b; Scott and Plückthun, 2013). It was applied in Plückthun’s lab and ultimately enabled structural determination of the functional NTS_1_ receptor variants expressed in the inner membrane of *E. coli* (Egloff et al., 2014b). After expression of an extensive DNA library of receptor variants in *E. coli,* the fluorescent ligands are bound to the receptors and the best expressers are enriched in FACS. This can be combined with subsequent radioligand binding to measure thermal stabilities (Dodevski and Plückthun, 2011) or used to assess detergent stabilities in a highly innovative method of cellular high-throughput encapsulation, solubilization and screening (Scott and Plückthun, 2013). In the later approach, each *E. coli* cell expressing a GPCR mutant is encapsulated with the detergent-resistant polysaccharide matrix. After detergent solubilization, a whole cellular content, including solubilized receptor and the corresponding plasmid DNA, stays inside the polysaccharide capsule. Binding of a fluorescently labeled ligand enables FACS enrichment with the most detergent-stable receptors and, at the same time, enrichment of the DNA carrying the related gene. The sorted genes are further amplified in PCR and subjected to new selection (and mutagenesis) rounds.

As we are getting more understanding of GPCR structures and effects of individual mutations, computational methods of thermostabilizing mutation predictions are under way (Bhattacharya et al., 2014).

### Other Point Mutations

*N*-glycosylation sites which are not required for protein expression and proper folding can be removed by changing the involved asparagine residue(s) to aspartate (Haga et al., 2012), glutamate (Rasmussen et al., 2007), glutamine (Kruse et al., 2012), alanine (Doré et al., 2011; Lebon et al., 2011b), glycine or serine (Zhang et al., 2012), or even cysteine (in order to form the engineered disulfide bridge; Xie et al., 2003). C116^3.27^L substitution in turkey β_1_-adrenoceptor (Parker et al., 1991; Warne et al., 2008) and M96^2.67^T/M98T substitutions in human β_2_-adrenergic receptor N-terminally fused with T4L (Zou et al., 2012) enhance functional expression in insect cells. The palmitoylation site in turkey β_1_-adrenoceptor was mutated to alanine to increase sample homogeneity for *in surfo* crystallization (Warne et al., 2009). Nevertheless, the equivalent modification was not needed for other GPCRs that were crystallized with not truncated palmitoylation sites and palmitoyl moieties were even identified in crystal structures of β_2_-adrenoceptor (Cherezov et al., 2007), 5-HT_2B_ receptor (Liu et al., 2013; Wacker et al., 2013) and in almost all bovine and squid rhodopsin structures. If a covalent ligand is available, a point mutation might be necessary to allow for the chemical reaction between the ligand and protein, as exemplified by β_2_-adrenoceptor, for which H93^2.64^C mutation has been generated (Rosenbaum et al., 2011). The point mutations were also applied to improve purification yield (Zhang et al., 2014b) or to facilitate crystallization (Fenalti et al., 2014). Of course, some point mutations were introduced only to study structure–function relationship of the modified receptors or to switch a receptor into particular conformational state (Wu et al., 2010; Standfuss et al., 2011; Deupi et al., 2012; Singhal et al., 2013).

### Fusion Partners

Soluble protein fusion partners inserted into a GPCR molecule can have a dual role. Primarily, they increase a hydrophilic surface of a GPCR chimera which is involved in intermolecular contacts in a crystal. Furthermore, a fusion partner inserted into a receptor loop can conformationally stabilize GPCR molecule and reduce molecular flexibility. Increased hydrophobicity and reduced flexibility can both facilitate and speed up crystallization. In general, fusion partners do not increase thermostability. Nevertheless, the chimeric constructs were exclusively used for crystallization in lipidic mesophases, so additional stabilization by point mutations was not necessary for most of them.

**FIGURE 5:**
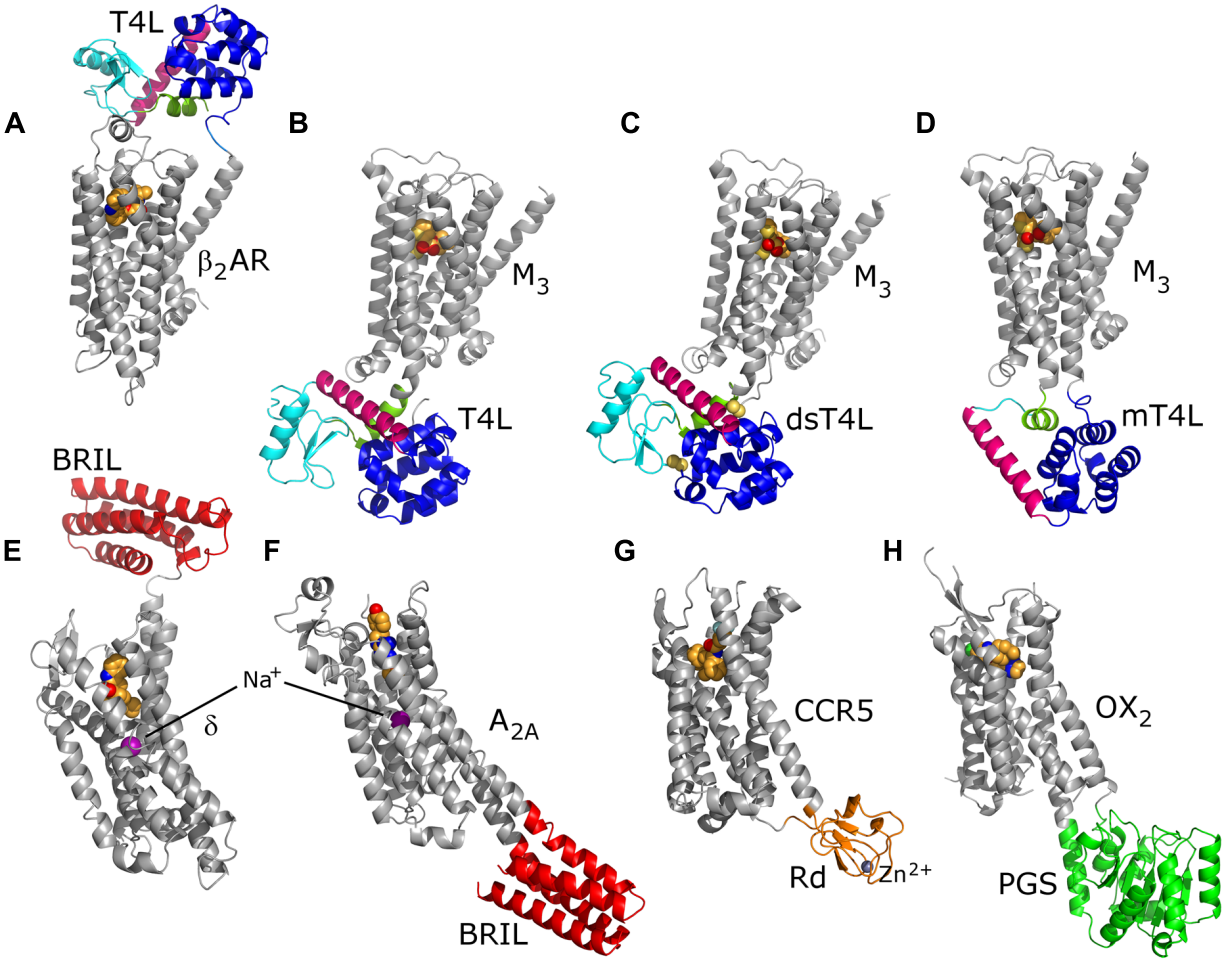
**Variety of fusion partners used for crystallization of GPCRs.** A flexible T4L molecule consists of an N-terminal (cyan) and a C-terminal lobe (blue) connected via a helix C (pink); helix A (green) precedes the N-terminal lobe **(A–D)**. Disulfide bonds (yellow spheres) decrease molecular flexibility in dsT4L **(C)**. On the other hand, molecular size of T4L was reduced by deleting the N-terminal lobe in mT4L **(D)**. The insertion sites of the fusion partners into ICL 3 are exactly the same for all three M_3_ receptor constructs **(B–D)**. Both T4L and BRIL (red) were used as N-terminal fusion partners **(A,E)**. Structures of the BRIL chimeras show clearly the sodium-ion allosteric site **(E,F)**. In the Rd (orange) chimera, a Zn^2+^ ion substitutes the naturally occurring Fe^2+/3+^ **(G)**. PGS (green) was identified as a fusion partner most recently **(H)**. For all structures, C atoms of the orthosteric ligands are represented as orange spheres. PDB IDs: 4GBR **(A)**, 4DAJ **(B)**, 4U14 **(C)**, 4U15 **(D)**, 4N6H **(E)**, 4EIY **(F)**, 4MBS **(G)** and 4RNB **(H)**.

The first such structure was that of human β_2_-adrenoceptor with the cysteine-free (C54T/C97A) mutant of T4L inserted instead of a large portion of ICL 3 (Cherezov et al., 2007). As always a case with fusion partners, T4L insertion point had to be extensively screened before finding the optimal chimeric construct (Rosenbaum et al., 2007). Insertion of T4L into ICL 3 of a GPCR was subsequently used for obtaining crystal structures of many class A GPCRs (**Table 2**; **Figure 5**). T4L was also attached at the truncated N-terminus of β_2_-adrenoceptor (Zou et al., 2012). Moreover, this construct was used for crystallization of β_2_-adrenoceptor in complexes with agonists and nanobody (Ring et al., 2013; Weichert et al., 2014) as well as for the pivotal complex with G_s_ protein (Rasmussen et al., 2011b; **Figure 1**). Class B CRF_1_ receptor (Hollenstein et al., 2013) and class C mGlu_5_ receptor (Doré et al., 2014) were both crystallized with T4L inserted into the ICL 2. Quite recently two new versions of T4L have been developed and used for crystallization of muscarinic M_3_ receptor (Thorsen et al., 2014). Namely, the original cysteine-free T4L contains two lobes connected with a flexible hinge. Such a flexible T4L structure is not an optimal feature for crystallogenesis. In a newly designed ‘disulfide-stabilized’ T4L (dsT4L) four mutations have been introduced to establish two disulfide bridges in order to reduce conformational flexibility. In ‘minimal’ T4L (mT4L), the whole N-terminal lobe was substituted by a small linker thus reducing both molecular size and flexibility (**Figure 5**).

**Table 2:** G protein-coupled receptors crystallized as chimeric proteins (sorted by the fusion partner and the attachment sites).

**T4L in ICL 3** β_2_-adrenoceptor (Cherezov et al., 2007; Hanson et al., 2008; Wacker et al., 2010; Rasmussen et al., 2011a; Rosenbaum et al., 2011) not thermostabilized variant of A_2A_ receptor expressed in insect cells (Jaakola et al., 2008; Xu et al., 2011b) D_3_ receptor (Chien et al., 2010) CXCR4 (Wu et al., 2010) H_1_ receptor (Shimamura et al., 2011; Shiroishi et al., 2011) M_2_ receptor bound to an antagonist (Haga et al., 2012) M_3_ receptor (Kruse et al., 2012) μ receptor (Manglik et al., 2012) κ receptor (Wu et al., 2012) mouse δ receptor (Granier et al., 2012) NTS_1_ receptor expressed in insect cells and crystallized in LCP (White et al., 2012) PAR1 (Zhang et al., 2012) S1P_1_ receptor (Hanson et al., 2012) FFA1 receptor (Srivastava et al., 2014)

**T4L in ICL 2** CRF_1_ receptor (Hollenstein et al., 2013) mGlu_5_ receptor (Doré et al., 2014)

**T4L at N-terminus** β_2_-adrenoceptor (Zou et al., 2012) β_2_-adrenoceptor in complex with G_s_ protein and nanobody (Rasmussen et al., 2011b) β_2_-adrenoceptor in complexes with agonist and nanobody (Ring et al., 2013; Weichert et al., 2014)

**mT4L in ICL 3** M_3_ receptor (Thorsen et al., 2014)

**dsT4L in ICL 3** M_3_ receptor (Thorsen et al., 2014)

**BRIL in ICL 3** A_2A_ receptor (Liu et al., 2012b) 5-HT_1B_ receptor (Wang et al., 2013a) 5-HT_2B_ receptor (Wacker et al., 2013) P2Y_12_ receptor (Zhang et al., 2014a) SMO (Wang et al., 2014; Weierstall et al., 2014)

**BRIL at N-terminus** NOP receptor (Thompson et al., 2012) glucagon receptor (Siu et al., 2013) SMO (Wang et al., 2013b, 2014) mGlu_1_ receptor (Wu et al., 2014) human δ receptor (Fenalti et al., 2014)

**Rd in ICL 3** CCR5 (Tan et al., 2013)

**PGS in ICL 3** OX_2_ receptor (Yin et al., 2015)

In addition to T4L, some other potential fusion partners were determined (Chun et al., 2012). The thermostabilized BRIL was utilized for crystallization of a number of GPCRs, either as N-terminally attached or substituting the ICL 3 (**Table 2**). In two instances where T4L or BRIL chimeras did not result in crystals, homogenous samples or crystallization constructs that are stable enough, Rd (Tan et al., 2013) and PGS (Yin et al., 2015) appeared as valid alternatives.

Several GPCRs have been crystallized as different chimeras or both with a fusion partner and without any fusion protein, either as a thermostabilized variant or in a complex with Fab fragment or nanobody. Examples for these are β_2_-adrenoceptor, A_2A_ receptor, NTS_1_ receptor, SMO and M_3_ receptor. This shows that different strategies in designing crystallization constructs can be equally successful. Crystallization of the ultra-thermostable mutant of turkey β_1_-adrenoceptor in LCP (Miller-Gallacher et al., 2014) demonstrated that a fusion partner is not required for LCP crystallization of GPCRs. It is worth noting that chimeras of A_2A_ receptor with BRIL inserted into a ICL 3 and that of human δ receptor with BRIL at the N-terminus both resulted in the highest-resolution GPCR crystal structures published so far (at 1.8 Å resolution), revealing the sodium-ion allosteric site (Liu et al., 2012b; Fenalti et al., 2014; **Figure 5**).

In designing the expression construct one should not forget to include tags important for expression and incorporation into the membrane, especially when native N-terminal part is absent from the construct or if one uses *E. coli* for expression. For example, cleavable hemagglutinin signal peptide significantly increased functional expression of β_2_-adrenoceptor in insect cells (Guan et al., 1992), therefore it was added to the majority of GPCR crystallization constructs expressed in insect cells. Some GPCRs showed higher expression in yeast when yeast α-factor secretion signal was included in the expression construct (Yurugi-Kobayashi et al., 2009). This was true for A_2A_ receptor (Hino et al., 2012), but not for H_1_ receptor for which functional expression was fivefold higher when expressed without α-factor signal sequence (Shiroishi et al., 2011). MBP at the N-terminus and TrxA at the C-terminus directed expression of rat NTS_1_ receptor into *E. coli* inner membrane (Egloff et al., 2014b), while having GST at the N-terminus of human CXCR1 enabled expression of this chemokine receptor as inclusion bodies in *E. coli* (Park et al., 2012b). Inclusion of GFP, as for H1 receptor expressed in *P. pastoris* (Shimamura et al., 2011; Shiroishi et al., 2011), facilitates characterization of a GPCR sample by measuring GFP fluorescence.

## Characterization

A construct for structural studies has to satisfy several criteria: (i) it should be expressed in sufficient amounts; (ii) it should be stable enough after solubilization; and (iii) it should make a homogenous sample after purification. To find the most suitable one, many constructs have to be characterized in a high-throughput manner.

Ligand-binding assays (reviewed in Fang, 2012) are usually methods of choice for measuring expression levels, receptor stability in different conditions or enrichment by purification. Because the ligand binds specifically to the receptor, it characterizes only molecules capable for ligand binding and excludes the misfolded ones. Moreover, it does not necessitate purifying sample, so it can be performed on whole cells, membrane preparations or detergent-solubilized GPCRs. Very sensitive radioligand-binding assays were mostly used for that purpose, but nowadays assays based on fluorescent ligands, as homogeneous time resolved fluorescence (Zwier et al., 2010) or fluorescence anisotropy titration (Huwiler et al., 2010), are becoming more in use. Alternatively, when labeled ligands are not available for the studied receptor, surface plasmon resonance spectroscopy can be applied instead to assess ligand-binding (Patching, 2014).

Including a fluorescent fusion tag, like GFP, can further facilitate analysis (Drew et al., 2006, 2008). Expression levels can directly be measured on whole cells or a detergent-solubilized sample and in-gel fluorescence, substituting a more traditional immunoblotting, gives additional information about size of the expressed GPCR molecule and its possible degradation. Here, we have to be careful, since it was shown that there is no correlation between fluorescence signal corresponding to total receptor expression and amount of functional receptor determined by radioligand binding (Shiroishi et al., 2012; Thomas and Tate, 2014). An alternative way of determining the amount of properly folded receptor molecules is by measuring cellular surface expression either with a fluorescently labeled ligand or with fluorescently labeled antibody that binds to an extracellular part of a receptor or to an engineered N-terminal tag (e.g., FLAG-tag). Both approaches have been used in high-throughput flow cytometry screening of GPCR variants (Hanson et al., 2007; Scott and Plückthun, 2013).

Homogeneity of the sample after solubilization can be monitored by FSEC (Kawate and Gouaux, 2006), either by utilizing intrinsic tryptophan fluorescence in purified samples or fluorescence of a fused GFP molecule, in which case a crude whole-cell lysates can also be used. By measuring a peak height for aliquots of the same sample incubated at different temperatures, FSEC can also be used to monitor thermostability of a GPCR sample (Hattori et al., 2012). Thermostability of a pure sample can be determined in the differential scanning fluorimetry based on the dye for which fluorescence increases after its covalent binding to the denatured protein in water solution (Alexandrov et al., 2008) or LCP (Liu et al., 2010).

Of course, purity of the final GPCR sample should always be examined in polyacrylamide gel electrophoresis and, if possible, the protein molecular weight and presence of posttranslational modifications checked by mass spectrometry (Ho et al., 2008). If applicable, the construct chosen for structural studies should also be characterized for its coupling with the cytosolic interacting partner (Thomsen et al., 2005; Siehler, 2008).

Flexibility of crystallization fusion constructs can be establish in a limited proteolysis assay (Rosenbaum et al., 2007). High-throughput FRAP was utilized to predict crystallizability of GPCR constructs in LCP (Cherezov et al., 2008; Xu et al., 2011a). Namely, rate of molecular diffusion of fluorescently labeled membrane proteins in LCP is positively correlated to its crystallogenesis, so LCP-FRAP can serve as efficient pre-crystallization screening.

## Special Considerations for NMR Studies

G protein-coupled receptors are challenging targets for solution NMR: (i) they need to be solubilized by detergents or other membrane-mimicking systems, which can easily increase their effective molecular weight to over 100 kDa, causing very broad lines and low signal amplitudes; (ii) internal receptor dynamics in the micro- to millisecond range may lead to further line broadening; (iii) GPCRs are largely α-helical and have very low spectral dispersion; (iv) many GPCRs are marginally stable in detergents; and (v) the introduction of isotopes is difficult in most GPCR expression systems that yield functional receptors. In particular, the latter point represents a serious limitation.

Each NMR active nuclei in the protein is a potential reporter of the local conformational changes and dynamics behavior of the protein. The more reporters we have the more complete picture about the receptor we can build, provided we can resolve and assign individual peaks. A particular attention deserves deuteration of the sample as it dramatically improves the relaxation behavior of the protein leading to sharper peaks and improved signal to noise ratio.

Depending on the expression system used, different labeling schemes can be employed, ranging from uniform labeling in *E. coli* to labeling single type of amino acids in eukaryotic expression systems to covalently attaching NMR-active labels after the expression and purification.

Uniform ^15^N, ^13^C, and ^2^H labeling can be achieved effectively in *E. coli*. Indeed, the only receptor structures solved by NMR so far have been obtained from proteins produced in *E. coli* or in an *E. coli* cell-free expression system: the bacterial GPCR-like sensory rhodopsin (Gautier et al., 2010) and proteorhodopsin (Reckel et al., 2011) studied by solution NMR as well as the human GPCR CXCR1 (Park et al., 2012a,b) studied by solid-state NMR. The *E. coli* production of the ^15^N, ^13^C labeled CB_2_ receptor and subsequent solid-state NMR studies have also been reported (Kimura et al., 2014). However, due to the requirement of the sample to be incorporated in the liposomes, the sensitivity of the experiment was insufficient to record resolved 2D spectra.

Although this remains to be explored, the GPCRs engineered by molecular evolution approach for increased stability and improved expression in *E. coli* (Sarkar et al., 2008; Dodevski and Plückthun, 2011; Schlinkmann et al., 2012a,b; Scott and Plückthun, 2013; Egloff et al., 2014b) may be very promising targets for NMR studies.

In contrast to the few receptors expressed in *E. coli*, the overwhelming majority of crystallized eukaryotic GPCRs have been obtained in functional form from eukaryotic expression systems, especially insect cells, which provide a more developed protein folding, modification and membrane insertion machinery. Isotope labeling is difficult in eukaryotic expression systems, since NMR-active isotopes need to be introduced as isotope-labeled amino acids or by non-native chemical modifications. So far, labeling by deuterium has been achieved for rhodopsin produced in a worm *C. elegans* (Salom et al., 2014), but not in more common eukaryotic expression systems, which severely limits the achievable resolution and sensitivity of NMR spectra.

Earlier NMR studies on rhodopsin were limited to using ^31^P at the phosphorylation sites and ^19^F probes covalently attached via cysteine (Klein-Seetharaman et al., 1999; Getmanova et al., 2004). Such probes offer favorable relaxation behavior and only one or very few observed signals for the ease of interpretation. The first GPCR to be labeled for NMR using single amino acids (glycine, lysine, or tryptophan) was also rhodopsin expressed in HEK293S cells (Eilers et al., 1999; Klein-Seetharaman et al., 2002, 2004). It is worth mentioning that in the case of lysine, one out of potentially eight backbone peaks was observed and assigned to the flexible C-terminus of the receptors.

Similar approaches were used to characterize the changes in dynamic behavior of the well-studied β_2_-adrenoceptor in response to ligand binding. Detection of ^19^F resonances from two trifluoroethanethiol-labeled cysteine residues located on the cytoplasmic side revealed a two-state equilibrium in slow chemical exchange (>2 ms) that is modulated by the type of ligand (Liu et al., 2012a). Agonists lead to conformational rearrangements in transmembrane helix 6 and 7, while arrestin-biased ligands strongly affect transmembrane helix 7.

The alternative to fluorine labels was to use favorable relaxation properties of methyl side chain groups of methionine. The labeled moiety was conjugated via chemical ligation to a specific location on the extracellular face of the receptor. Shifts in resonances of one ^13^C-methyl-tagged lysine side chain in β_2_-adrenoceptor revealed an allosteric coupling from the ligand to the extracellular surface (Bokoch et al., 2010). More recently, all methionines in the protein were labeled by adding ^13^C-methyl methionine to the drop-out media during expression in insect cells (Kofuku et al., 2012; Nygaard et al., 2013). The number of methionines was reduced by mutagenesis to three or four to obtain interpretable spectra, which showed chemical shift changes that depended on ligand efficacy, correlated between the ligand binding site and the intracellular side, and were sensitive to the binding of a G-protein-mimetic antibody (Nygaard et al., 2013).

The yeast expression system could be used to produce protein labeled for NMR. Potentially, even deuteration, at least partial, is possible. Unfortunately, as only A_2A_ and H_1_ receptors were expressed in *P. pastoris* for crystallographic studies (Shimamura et al., 2011; Singh et al., 2012), no reports about expression of labeled receptors are published.

Recently, Salom et al. (2014) reported triple ^15^N, ^13^C, and ^2^H labeling of rhodopsin expressed in transgenic *C. elegans*. This approach might find broader application in expression of other GPCRs for NMR studies.

## Conclusion

Overall, large-scale production of GPCRs for crystallographic studies is very well established nowadays. Insect cells are used mostly, but other expression systems might play more important role in the future. Mild detergents, like DDM or LMNG, mixed with CHS and combined with high-affinity ligands generally provide enough receptor stability for LCP crystallization. Also, modification of GPCRs by protein engineering and use of antibodies as crystallization chaperones increases their crystallizability. Unfortunately, it is still not possible to design from scratch a protein construct that will give high-quality crystals. Therefore, high-throughput experimental screening of large number of potential crystallization constructs is unavoidable for now.

On the other hand, NMR of GPCRs is a very promising area of research that remains challenging due to the difficulties in production of milligram amounts of isotopically labeled receptor. With the exception of a few receptors which could be produced fully labeled in *E. coli*, studies will be limited to the detection of a few specifically labeled amino acids or chemically introduced groups. Certainly, the favorable relaxation properties of methyl groups and fluorine already allowed obtaining valuable insights in the dynamic properties of GPCRs. Potentially, the backbone NMR studies, which are inherently more sensitive to the conformational changes then side chains, will give us richer information about the dynamics of the receptors. Of course, additional challenges such as long-term protein stability, minimizing the size of the detergent micelle, and potentially restricting conformational flexibility of the receptors and improvements in the sensitivity of the NMR data collection methods will have to be addressed to make progress in the NMR studies of GPCR dynamics.

## Authors’ Note

While the article was under review, the crystal structures of human CXCR4 in complex with a viral chemokine antagonist vMIP-II (Qin et al., 2015), and of human cytomegalovirus GPCR homolog US28 in complex with the chemokine domain of human CXC3CL1 (Burg et al., 2015) were published. The T4L-fused CXCR4 and vMIP-II – both with a cysteine point mutation – were co-expressed in insect Sf9 cells and trapped in the complex by an engineered disulfide bond between the two binding partners (Qin et al., 2015). Two different US28 constructs and the chemokine domain of CXC3CL1 were expressed separately in mammalian HEK293S GnTI- cells using BacMam baculovirus transduction. A ternary complex between the chemokine domain, the N- and C-terminally truncated US28 and an alpaca nanobody gave better diffracting crystals in comparison to the full-length US28 in complex with the chemokine domain only (Burg et al., 2015).

## Conflict of Interest Statement

The authors declare that the research was conducted in the absence of any commercial or financial relationships that could be construed as a potential conflict of interest.

## Abbreviations

7TM: seven transmembrane α-helical
BRIL: apocytochrome *b*_562_RIL
C8E4: tetraethylene glycol monooctyl ether
CHAPS: 3-[(3-cholamidopropyl)dimethylammonio]-1-propanesulfonate
CHAPSO: 3-([3-cholamidopropyl]dimethylammonio)-2-hydroxy-1-propanesulfonate
CHS: cholesteryl hemisuccinate
CMA: carboxymethylaspartate
CMC: critical micelle concentration
ConA: concanavalin A
DDG: *n*-dodecyl-β-d-glucopyranoside
DDM: *n*-dodecyl-β-d-maltopyranoside
DG: *n*-decyl-β-d-glucopyranoside
DM: *n*-decyl-β-d-maltopyranoside
DMPC: 1,2-dimyristoyl-*sn*-glycero-3-phosphocholine
dsT4L: ‘disulfide-stabilized’ T4 lysozyme
FACS: fluorescence-activated cell sorting
FRAP: fluorescence recovery after photobleaching
FSEC: fluorescence-detection size-exclusion chromatography
GFP: green fluorescent protein
GST: glutathione *S*-transferase
GPCR: G protein-coupled receptor
HEGA-10: *n*-decanoyl-*N*-hydroxyethylglucamide
HPTO: *n*-heptane-1,2,3-triol
HRV: human rhinovirus
HTG: *n*-heptyl-β-d-thioglucopyranoside
ICL: intracellular loop
IMAC: immobilized metal-ion affinity chromatography
LCP: lipidic cubic phase
LDAO: lauryldimethylamine-oxide
LMNG: lauryl-maltose-neopentyl glycol
LMPG: 1-myristoyl-2-hydroxy-*sn*-glycero-3-[phospho-rac-(1-glycerol)]
MBP: maltose-binding protein
mT4L: ‘minimal’ T4 lysozyme
NG: *n*-nonyl-β-d-glucopyranoside
NMR: nuclear magnetic resonance
NTA: nitrilotriacetic acid
OG: *n*-octyl-β-d-glucopyranoside
OTG: *n*-octyl-β-d-thioglucopyranoside
PCR: polymerase chain reaction
PGS: the catalytic domain of *Pyrococcus abyssi* glycogen synthase
Rd: rubredoxin
ROS: rod outer segment
SEC: size-exclusion chromatography
T4L: T4 lysozyme
TEV: tobacco etch virus
TrxA: thioredoxin A.
